# The *Ustilago maydis* AA10 LPMO is active on fungal cell wall chitin

**DOI:** 10.1128/aem.00573-23

**Published:** 2023-09-13

**Authors:** Roseline Assiah Yao, Jean-Lou Reyre, Ketty C. Tamburrini, Mireille Haon, Olivier Tranquet, Akshay Nalubothula, Saumashish Mukherjee, Sophie Le Gall, Sacha Grisel, Sonia Longhi, Jogi Madhuprakash, Bastien Bissaro, Jean-Guy Berrin

**Affiliations:** 1 INRAE, Aix Marseille Univ, UMR 1163 Biodiversité et Biotechnologie Fongiques (BBF), Marseille, France; 2 IFP Energies Nouvelles, Rueil-Malmaison, France; 3 CNRS, Aix Marseille Univ, UMR 7257 Architecture et Fonction des Macromolécules Biologiques (AFMB), Marseille, France; 4 INRAE, Aix Marseille Univ, 3PE Platform, Marseille, France; 5 Department of Plant Sciences, School of Life Sciences, University of Hyderabad, Hyderabad, Telangana, India; 6 INRAE, UR1268 BIA, Nantes, France; 7 INRAE, PROBE Research Infrastructure, BIBS Facility, Nantes, France; Chalmers University of Technology, Gothenburg, Sweden

**Keywords:** filamentous fungi, fungal cell wall, lytic polysaccharide monooxygenase, chitinase, *Ustilago maydis*, plant pathogen, remodeling

## Abstract

Lytic polysaccharide monooxygenases (LPMOs) can perform oxidative cleavage of glycosidic bonds in carbohydrate polymers (e.g., cellulose, chitin), making them more accessible to hydrolytic enzymes. While most studies have so far mainly explored the role of LPMOs in a (plant) biomass conversion context, alternative roles and paradigms begin to emerge. The AA10 LPMOs are active on chitin and/or cellulose and mostly found in bacteria and in some viruses and archaea. Interestingly, AA10-encoding genes are also encountered in some pathogenic fungi of the Ustilaginomycetes class, such as *Ustilago maydis*, responsible for corn smut disease. Transcriptomic studies have shown the overexpression of the AA10 gene during the infectious cycle of *U. maydis*. In fact, *U. maydis* has a unique AA10 gene that codes for a catalytic domain appended with a C-terminal disordered region. To date, there is no public report on fungal AA10 LPMOs. In this study, we successfully produced the catalytic domain of this LPMO (*Um*AA10_cd) in *Pichia pastoris* and carried out its biochemical characterization. Our results show that *Um*AA10_cd oxidatively cleaves α- and β-chitin with C1 regioselectivity and boosts chitin hydrolysis by a GH18 chitinase from *U. maydis* (*Um*GH18A). Using a biologically relevant substrate, we show that *Um*AA10_cd exhibits enzymatic activity on *U. maydis* fungal cell wall chitin and promotes its hydrolysis by *Um*GH18A. These results represent an important step toward the understanding of the role of LPMOs in the fungal cell wall remodeling process during the fungal life cycle.

**IMPORTANCE:**

Lytic polysaccharide monooxygenases (LPMOs) have been mainly studied in a biotechnological context for the efficient degradation of recalcitrant polysaccharides. Only recently, alternative roles and paradigms begin to emerge. In this study, we provide evidence that the AA10 LPMO from the phytopathogen *Ustilago maydis* is active against fungal cell wall chitin. Given that chitin-active LPMOs are commonly found in microbes, it is important to consider fungal cell wall as a potential target for this enigmatic class of enzymes.

## INTRODUCTION

Lytic polysaccharide monooxygenases (LPMOs) are monocopper enzymes that catalyze the oxidative cleavage of glycosidic bonds in carbohydrate polymers. Their discovery has been a major breakthrough in the understanding of the microbial enzymatic mechanisms involved in the degradation of natural recalcitrant polymers, including cellulose and chitin (1–3). LPMOs can act at the surface of polysaccharides, in synergy with other oxidoreductases (4) and glycoside hydrolases (GHs), to overcome polymers recalcitrance factors (such as crystallinity), thereby boosting bioconversion yields (5–7). The unique catalytic properties of LPMOs make them of utmost interest for different types of applications, such as the valorization of lignocellulosic biomass for the production of bioproducts (e.g., biofuels) (8, 9) or bio-based materials (10–12). So far, most studies have explored the role of LPMOs from a biotechnology perspective. Excitingly, studies reported over the past few years have shown that LPMOs are present across nearly all kingdoms of life, encompassing thus a wide range of biological contexts, and pointing at the emergence of new roles and paradigms (13).

LPMOs are classified into eight auxiliary activity (AA) families in the carbohydrate-active enzymes (CAZy) database: AA9–AA11 and AA13–AA17 (13, 14). Although their substrate specificity varies among AA families, they are mostly active on cellulose, chitin, xylan, starch, or pectin. The AA10 family is the most taxonomically diverse family with sequences originating from bacteria (15–23), viruses (24), and archaea (25). Interestingly, the presence of AA10s goes beyond the prokaryotic domain of life, as they have recently been shown to be present in some plants (ferns) (26, 27) and in some pathogenic fungi. All AA10 LPMOs hitherto characterized have been found to be active on chitin (C1-oxidizers) and/or cellulose (C1- and C1/C4-oxidizers) (1, 3, 16). Of note, AA10s are often tested on model substrates such as shrimp/crab chitin or pretreated cellulose, and very little is known on their actual biological substrate. The identification of the latter is crucial to get insight into the biological function of a given LPMO but such approaches are challenging and require integration of the physiology of the living organism. A few attempts have been made along this line. Recent studies have notably shown the role of AA10 LPMOs in the chitin oxidative metabolism of a marine bacterium (28), in the virulence of pathogens (29–31), or even in the remodeling of bacterial cell wall (32).

*Ustilago maydis* is a biotrophic parasite (smut fungus) that depends on living tissue for proliferation and development in maize. This ubiquitous pathogen is also a well-established model organism for the study of plant-microbe interactions (33, 34). Compared to fungal saprotrophs and some plant pathogens, the *U. maydis* genome contains a small set of CAZymes (35) with 107 GHs and 23 AAs. While studying CAZymes from *U. maydis* (36, 37), we noted the unusual presence of a unique AA10-encoding gene in its genome (*Um*AA10), the function of which remains unknown. Interestingly, digging into transcriptomic data collected during *U. maydis* plant infection cycle (38), we noted that this *Um*AA10-encoding gene is overexpressed. Here, we managed to produce *Um*AA10 in a heterologous system and characterized it with model substrates and a more biologically relevant substrate prepared from *U. maydis* mycelium. We also probed the concerted action of *Um*AA10 together with a GH18 chitinase from *U. maydis* (*Um*GH18A). Our results, analyzed in light of previously published data in the context of *in planta* infection, provide hints on the biological function of *Um*AA10.

## RESULTS

### Bioinformatics analysis

**Fig 1:**
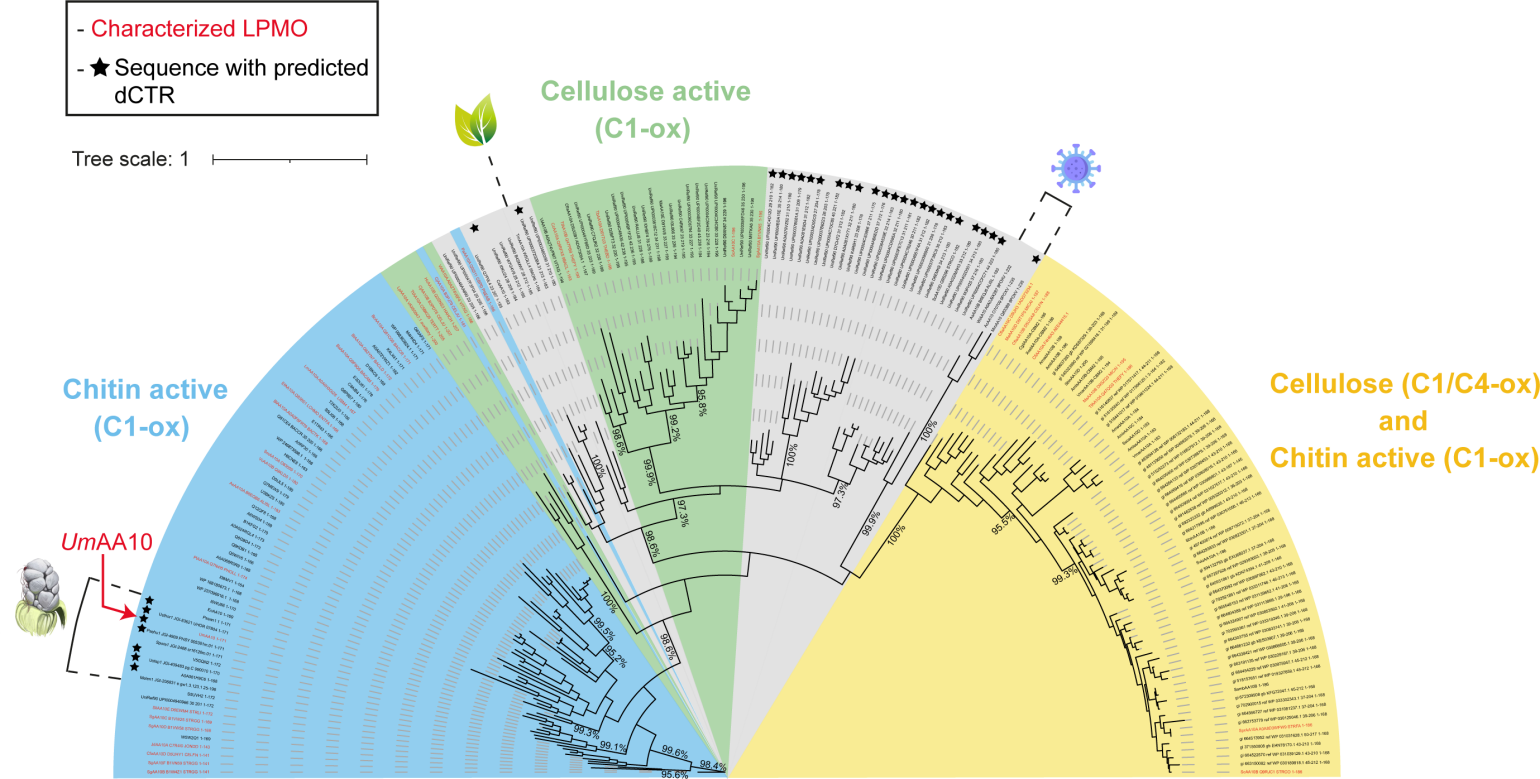
Phylogenetic tree of AA10s. The phylogenetic tree was generated based on a multiple alignment of 197 sequences (AA10 catalytic domain only). The predicted substrate specificity is based on the presence of characterized AA10 LPMOs (in red) within each clade. The substrate specificity of clades highlighted in gray has not been determined yet. AA10 sequences with a predicted dCTR are highlighted with a black star.

To get insight into the putative function of *Um*AA10, we built a phylogenetic tree using 197 AA10 amino acids’ sequences from bacteria, eukaryotes (fungi and plant), and viruses (Fig. 1). All fungal AA10 LPMOs (including *Um*AA10) cluster together within a larger clade of bacterial AA10s, some of which have been biochemically characterized as chitin-active with C1 regioselectivity. This observation could indicate horizontal gene transfer (HGT) events between bacteria and fungi (see Discussion). Another interesting observation is the presence of a predicted intrinsically disordered C-terminal region (dCTR) in *Um*AA10. dCTRs are regions of unknown function encountered in most LPMO families, and which occur in ~8% of AA10 sequences (39). Interestingly, while most Ustilaginomycetes AA10s display a dCTR, they are distantly related to bacterial AA10-dCTRs. More detailed analysis of these dCTR tails could help in finding common features among fungal AA10s or among AA10s from different species. To the best of our knowledge, none of these microbial AA10s bearing a dCTR have been characterized to date.

**Fig 2:**
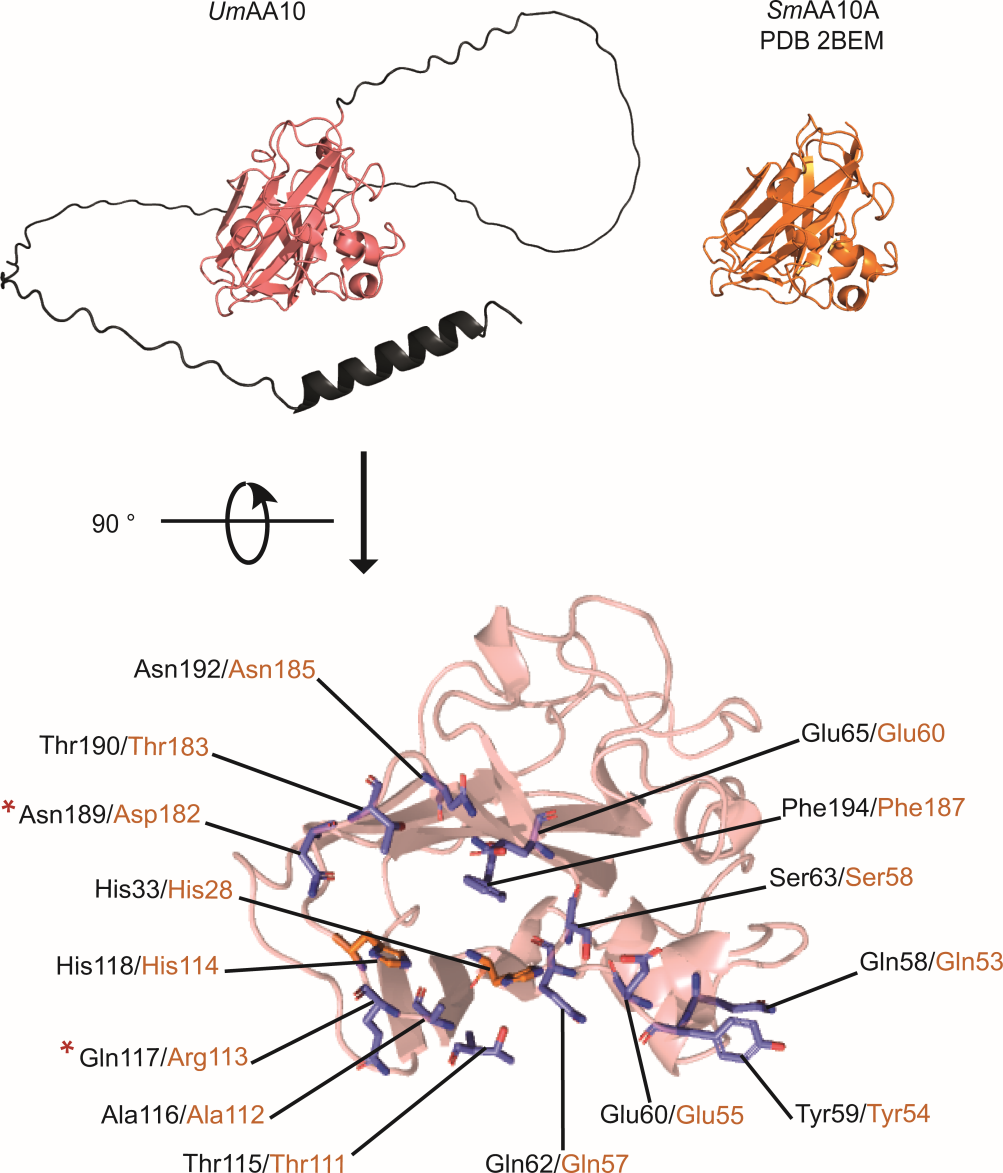
Structural analysis of *Um*AA10. (A) Overview of the predicted 3D structure (AlphaFold2) of *Um*AA10 and of the crystal structure of *Sm*AA10A [PDB 2BEM; see reference (15)]. The catalytic domain of the predicted *Um*AA10 structure is shown in salmon and the C-terminal extension in black. (B) *Um*AA10 predicted substrate-binding residues (shown as sticks) based on those identified in *Sm*AA10A. *Um*AA10 residues (labeled in black) differing from the corresponding residues occurring in *Sm*AA10A (labeled in orange) are marked by a red star.

The *Um*AA10 sequence is composed of 326 amino acids, with a signal peptide (1–32), an AA10 LPMO catalytic domain (cd, 33–203), and a dCTR (204–326). The 3D structure of *Um*AA10 (Fig. 2A), predicted using AlphaFold2 (40, 41), shows that the catalytic domain displays an immunoglobulin-like β-sandwich fold, typical of LPMOs, with a planar surface exposing the active site formed by two His residues (H33 and H118) coordinating the copper atom. Residues involved in chitin binding and LPMO activity in the archetypal AA10 LPMO from *Serratia marcescens*, *Sm*AA10A [PDB 2BEM (15)], also known as CBP21, have been previously identified using nuclear magnetic resonance (NMR) (42), site-directed mutagenesis (15) and *in silico* quantum mechanics and molecular dynamics (43). Some of these *Sm*AA10A key residues are also conserved in *Um*AA10, i.e., Gln58, Tyr59, Glu60, Gln62, Ser63, Thr115, Ala116, His118, Asn189, and Asn192 (Fig. 2B). Moreover, the “TAXH” motif (Thr115, Ala116, and His118), conserved in all chitin-active AA10 LPMOs (44), is also found in *Um*AA10, where X = Gln117. This Gln residue could indicate a preference of *Um*AA10 for α-chitin. Indeed, it was previously shown that the equivalent residue in *Sm*AA10A (Arg113) is important for its binding preference for β-chitin (44). Altogether, these bioinformatic observations suggest that *Um*AA10 could target chitin.

As mentioned above, *Um*AA10 displays a long region (120 residues) predicted to be intrinsically disordered by three different predictors, i.e., MobiDB-lite integrated in InterPro, IUPred2A, and AlphaFold pLDDT (Fig. S1) (41, 45, 46). The length of this dCTR is close to that of the other dCTRs identified in AA10s (median value = 123 residues; Fig. S1). The dCTR of *Um*AA10, enriched in Ser (35%), Gly (11%), Ala (10%), Arg (8%), and Thr (8%), is overall hydrophilic with a positively charged patch (Arg-rich) at the C-terminus. Charged residues and distribution of residues of opposite charge along the sequence are one of the main determinants of the conformational properties of intrinsically disordered regions (47–49). In addition, they could be involved in protein-protein or protein-membrane electrostatic interactions. Interestingly, the last 47 residues of the *Um*AA10-dCTR are also predicted by ANCHOR2 (46) to be a disordered binding site. The alignment of the last 50 residues of dCTRs from 30 AA10s revealed some degree of conservation and the presence of a patch of highly positive charged residues at the end of the sequence [see Fig. S2 and reference (50)], which might suggest a common, yet unelucidated, role across fungal AA10s.

### Recombinant production of *Um*AA10

To investigate the biochemical properties of *Um*AA10, we attempted its heterologous production in the yeast *Pichia pastoris*. Unfortunately, even after several attempts, the recombinant production of *Um*AA10 (in its full-length form) failed, probably due to the disordered nature of the long C-term extension. Therefore, we decided to produce only the catalytic domain (hereafter referred to as *Um*AA10_cd). Interestingly, we observed upon *Um*AA10_cd recombinant production a striking difference in cells’ sedimentation and cell shape/size (Fig. S3). Addition of ethylenediaminetetraacetic acid (EDTA) to the culture (to potentially inactivate *Um*AA10_cd by copper chelation) restored a “normal” sedimentation behavior of *P. pastoris* cells and partially restored cell shape/size (Fig. S3). However, the recombinant production yield was not further improved (~0.35 mg of pure protein per liter of culture). After a two-step purification by nickel-affinity and size-exclusion chromatography, *Um*AA10_cd was purified to homogeneity. It displayed an apparent molecular weight of ~20 kDa on SDS-PAGE (Fig. S4) in good agreement with the theoretical one (19.5 kDa). After copper loading, *Um*AA10_cd contains ~1.4 copper atom per protein molecule, as determined by inductively coupled plasma mass spectrometry (ICP-MS).

### Substrate specificity of *Um*AA10_cd

**Fig 3:**
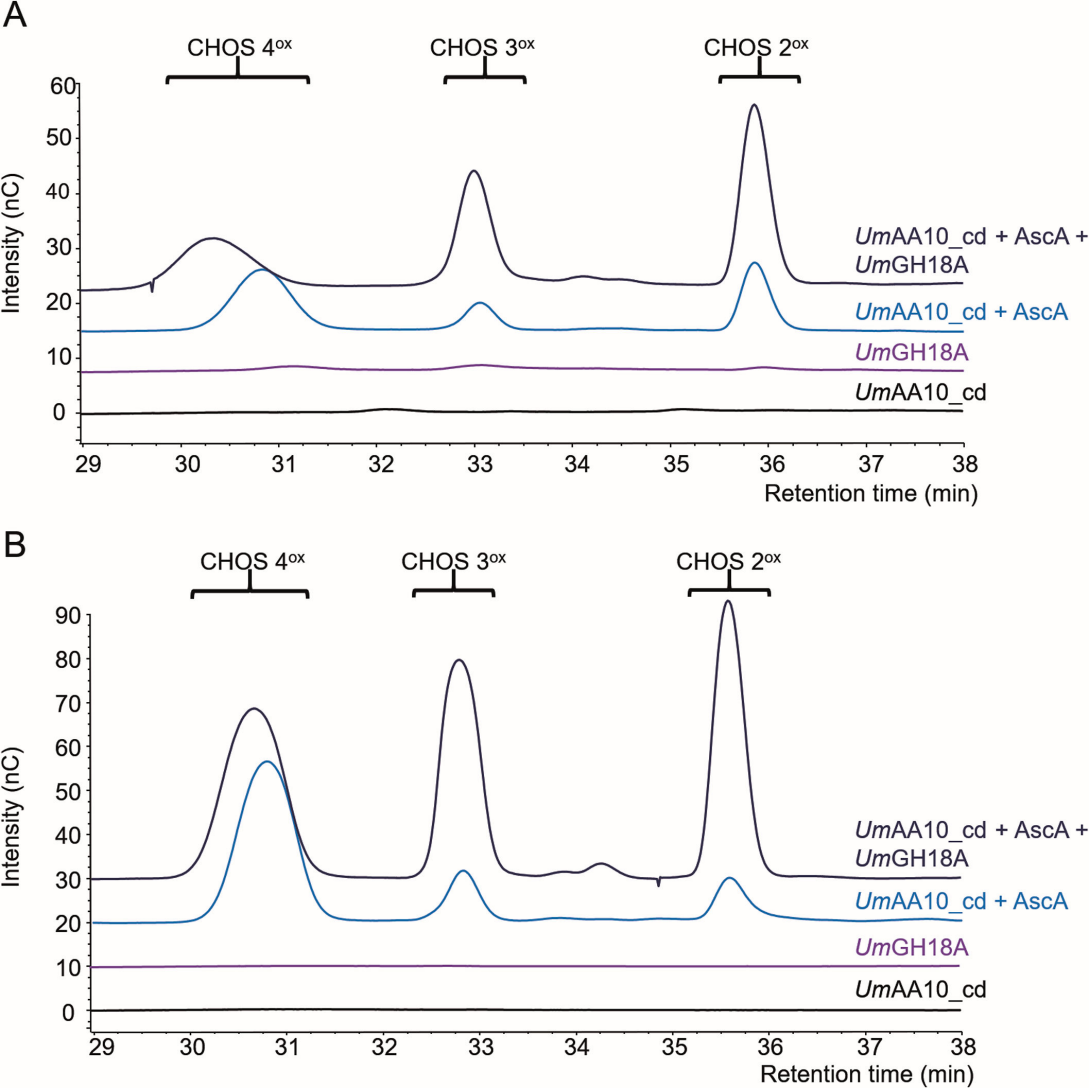
Activity of *Um*AA10_cd on chitin. High-performance anion-exchange chromatography-pulsed amperometric detection chromatograms of oxidized chitooligosaccharides (CHOS) released from α-chitin (A) and β-chitin (B) by either *Um*GH18A (0.1 µM) or *Um*AA10_cd (1 µM), alone or combined. Reactions were carried out in sodium phosphate buffer (50 mM, pH 6.0) under stirring (1,000 rpm) at 30°C for 24 h. For each condition, three independent biological replicates were carried out and, for the sake of clarity, only one replicate is shown.

Before assessing the substrate specificity of *Um*AA10_cd, we first tested its ability to reduce O_2_ to H_2_O_2_ in the presence of an appropriate reducing agent (51). Using ascorbic acid (AscA) as a reductant, we found that *Um*AA10_cd produces H_2_O_2_ at a rate of 3.8 × 10^−4^ s^−1^, which reflects a relatively average oxidase activity, compared to other LPMOs (4) (Fig. S5). To investigate the substrate specificity of *Um*AA10_cd, we tested several relevant model substrates of AA10 LPMOs, i.e., α-chitin, β-chitin, crystalline cellulose (Avicel), and phosphoric acid swollen cellulose (PASC), with AscA being consistently used as a reducing agent. We observed the release of several products corresponding to C1-oxidized chito-oligosaccharides (DP2–DP4) from α- and β-chitin (Fig. 3A and B). No activity was detected on cellulose (Fig. S6). These results are in line with our bioinformatic predictions (Fig. 1). To deepen our knowledge on *Um*AA10_cd, we further tested its activity in synergy with a GH18 chitinase. Indeed, many studies have demonstrated the synergistic action of LPMOs and GHs in the degradation of plant polysaccharides (52, 53), chitin (1), and other glycans such as peptidoglycan (32). In the context of our study, we hypothesized that *Um*AA10_cd could work in synergy with one of the GH18 from *U. maydis* for the degradation of chitin. The genome of *U. maydis* contains three genes coding for GH18s: *UMAG_02758* (*Um*GH18A), *UMAG_10419* (*Um*GH18B), and *UMAG_06190* (*Um*GH18C) [respectively, *cts2*, *cts1*, and *cts3* in reference (54)]. While *Um*GH18A and *Um*GH18C harbor a signal peptide*, Um*GH18B is predicted to be secreted by an unconventional pathway (55). In this study, we focused our attention on *Um*GH18A, which was previously shown to be active during yeast growth (54) and upregulated during maize infection (38). After successful production in *P. pastoris* and purification to homogeneity (Fig. S4), we evaluated its activity toward chitin. *Um*GH18A displayed optimal activity at 40°C and pH between 4 and 5 (Fig. S7), and exhibited *exo*-acting hydrolytic activity on α- and β-chitin, releasing chitobiose (CHOS 2) as a major product (Fig. S8). Interestingly, *Um*GH18A seems to be more active on β-chitin.

It is now clearly established that the preferred co-substrate of LPMOs during the oxidative cleavage of polysaccharides is H_2_O_2_ (56, 57). LPMOs have also been reported to exhibit a peroxidase activity in the presence of specific organic reductants and H_2_O_2_ (58). However, using reaction conditions described by Bissaro et al. (44) and Breslmayr et al. (58) (see Materials and Methods for further details), we could not detect any significant peroxygenase activity (on α-chitin) or peroxidase activity (on 2,6-dimethoxyphenol [DMP]) (data not shown). This result is possibly due to an extra-sensitivity of *Um*AA10_cd to H_2_O_2_ as it has been shown that LPMOs are subject to oxidative inactivation (56, 59, 60), a phenomenon that depends on various reaction conditions.

#### Enzymatic assays on a biologically relevant substrate

**Fig 4:**
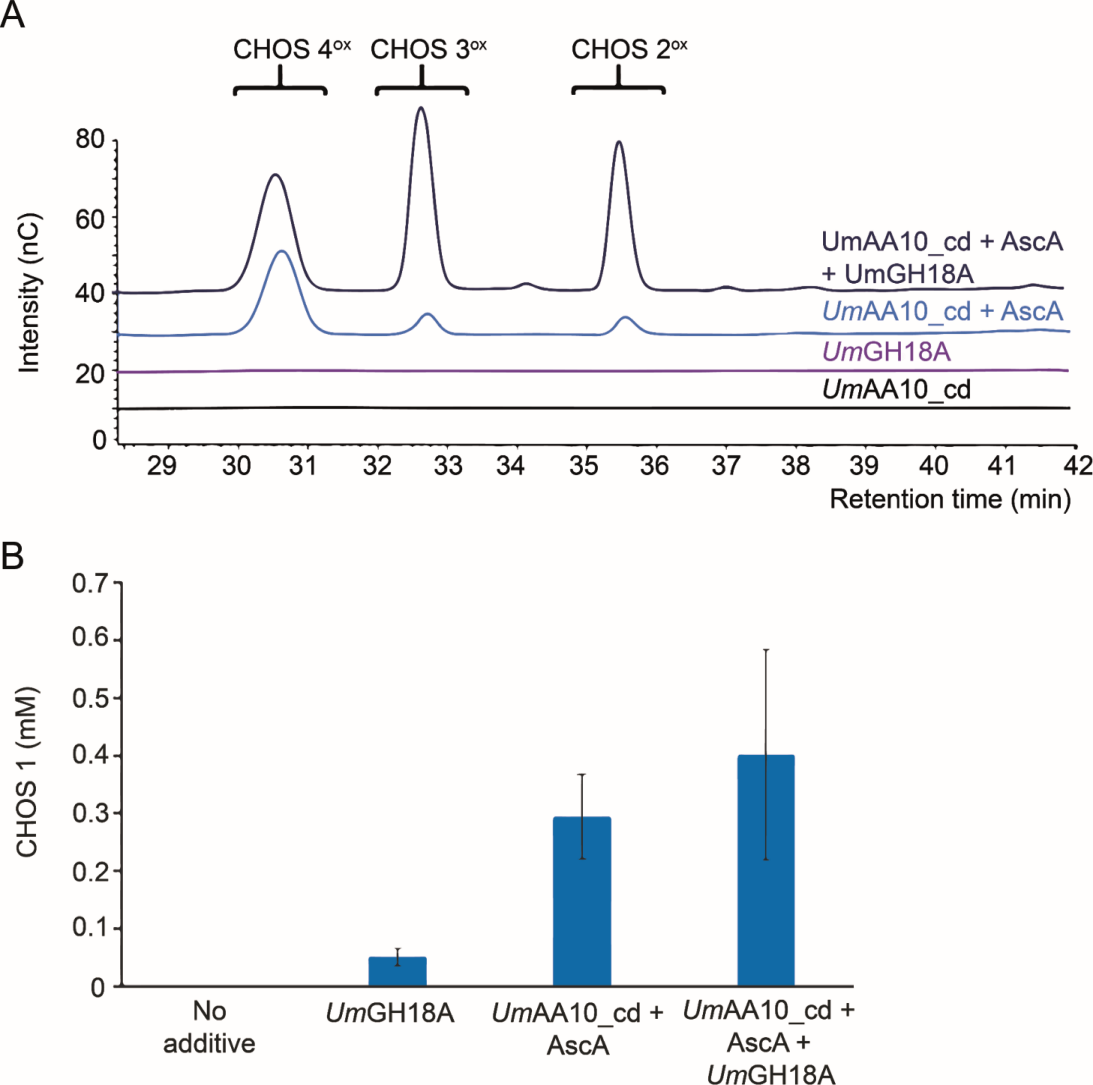
Activity of *Um*AA10_cd on *U. maydis* fungal cell wall chitin. (A) The graph shows high-performance anion-exchange chromatography-pulsed amperometric detection chromatograms of oxidized CHOS released from *Um*FCW (10 g/L) by either *Um*GH18A (0.1 µM) or *Um*AA10_cd (1 µM) and AscA (1 mM), alone or combined. (B) Quantification of total soluble products (from the reaction described in panel A). A GH20 chitobiase from *Serratia marcescens* (*Sm*GH20) was used to degrade the mixtures of CHOS to GlcNAc (CHOS 1) and GlcNAc-GlcNAc1A (CHOS 2^ox^), as described by Loose et al. (17). All reactions were carried out in sodium phosphate buffer (50 mM, pH 6.0) under stirring (1,000 rpm) at 30°C for 24 h. Data points show average values and error bars correspond to standard deviations from three independent biological replicates.

To go further into the biochemical characterization *Um*AA10_cd, we aimed at testing it on a more biologically relevant substrate. As plant cell wall is devoid of chitin, the most obvious source of chitin in the environment of *U. maydis* is its own cell wall, which is remodeled during pathogenesis. We therefore prepared a fraction containing chitin from *U. maydis* mycelium produced at the filamentous stage (see Materials and Methods). The presence of chitin in the alkali-insoluble fraction was confirmed by compositional analysis and by fluorescence microscopy using the labeled wheat germ agglutinin (WGA) lectin (Fig. S9), a reagent commonly used to study chitin and chitin-like molecules (61). Taking advantage of this *U. maydis* fungal cell wall fraction (*Um*FCW), we were able to assay the activity of *Um*AA10_cd on a more natural form of chitin. As previously shown on model chitin, *Um*AA10_cd was found to exhibit enzymatic activity on *Um*FCW with the release of C1-oxidized CHOS. We also provide experimental evidence that *Um*AA10_cd acts in concert with *Um*GH18A on *Um*FCW increasing the overall release of soluble CHOS (Fig. 4).

## DISCUSSION

In this study, we demonstrate, using complementary approaches, that *Um*AA10 is an LPMO that oxidatively cleaves *U. maydis* FCW extracts containing chitin, and that acts together with a GH18 chitinase from *U. maydis* (*Um*GH18A). We would like to emphasize that this study reports the first characterization of a fungal LPMO belonging to the AA10 family and the first biochemical evidence of LPMO activity toward FCW chitin. The appearance of AA10 LPMOs in the fungal kingdom may be ascribed to HGT. In fact, HGT between bacteria and eukaryotes has been documented for cell wall degrading enzymes (62, 63) and it is well known that HGT can provide a new function or replace a functional loss in the recipient organism, allowing adaptation to its environment (64). It would therefore be interesting to further investigate these events by focusing on the fungal members of the AA10 family.

**Fig 5:**
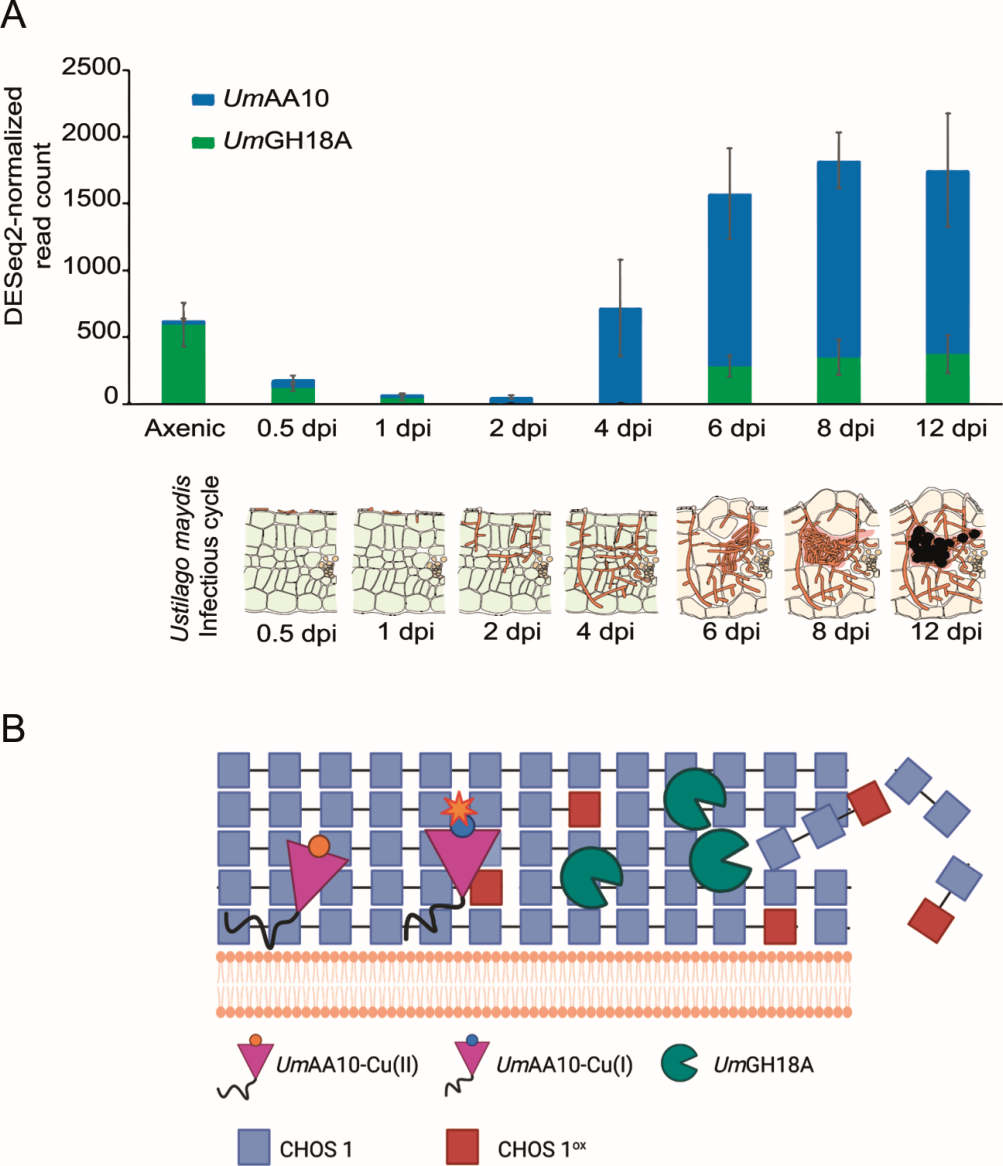
Putative function of *Um*AA10 *in vivo*. (A) Normalized expression of genes coding for *Um*AA10 and *Um*GH18A during corn infection, illustrated with data adapted from Lanver et al. (38). Under each bar graph, is presented the corresponding *U. maydis* infection cycle stage. dpi: day post-infection. (B) Schematic view of the proposed cooperation between *Um*GH18A and *Um*AA10 *in vivo*. The figure was created using BioRender.com.

On the basis of transcriptomic data from Lanver et al. (38), we know that the gene encoding *Um*AA10 is overexpressed during advanced stages of maize infection, i.e., between 4 and 12 days post-infection (dpi) (Fig. 5A), when we observe major morphological changes that entail a fast and massive rearrangement of *U. maydis* cell wall with modification of FCW polysaccharides, including chitin. The FCW is a key adaptable scaffold for the survival of the fungus notably during host infection (65). The structural and mechanical properties of the FCW allow the fungus to resist the turgor pressure necessary for polar growth (66) and to withstand environmental stresses (67, 65, 68). While the mechanisms involved in this cell wall biochemical changes are still poorly known, some CAZymes from *U. maydis,* such as chitinases (54) and chitin deacetylases (69), have been shown to be involved in FCW modification and virulence. However, to date the role of oxidative enzymes in FCW modification has not been deeply investigated in a chitinolytic context. Of note, we recently characterized two CAZymes from *U. maydis* that act in synergy on β-glucans, namely a glucanase (*Um*GH16_1A) active on β-1,3-glucans branched with short β-1,6 substitutions and a dehydrogenase (*Um*AA3_2A) active on β-1,3 and β-1,6-gluco-oligosaccharides released by the former (37). As these substrates are also FCW components, we believe these enzymes could play a role in FCW remodeling.

In a different context, it was recently discovered that a chitin-active AA11 LPMO from *Neurospora crassa* (CWR-1) is involved in cell-cell allorecognition and cell wall dissolution during cell fusion (70). Additionally, another fungal LPMO from the AA9 family (*Cn*CEL1), which harbors a dCTR and is localized at the cell surface, has been suggested to be involved in cell wall integrity and cell cycle of the human pathogen *Cryptococcus neoformans* (71). Beyond the fungal kingdom, a recent study similarly revealed the involvement of an AA10 LPMO from *Streptomyces coelicolor* (named as LpmP by the authors) in cell wall remodeling (32). This LPMO is retained in the bacterial cell wall and facilitates peptidoglycan degradation by lysozyme. Together with our findings, it suggests that the remodeling of microbial cell wall polysaccharides by LPMOs may be widespread in nature. Considering the robustness of FCW chitin (72) and while *U. maydis* displays no AA11 LPMO and a unique chitin-active AA10 LPMO, we can hypothesize that *Um*AA10, in addition to *U. maydis* chitinases, could be involved in the modification of chitin upon cell wall remodeling and morphological changes during maize infection (Fig. 5). Despite the need for functional *in cellulo* validation in *U. maydis*, this proposed biological role is supported by the presence of a dCTR that could anchor *Um*AA10 in the FCW and by the phenotype observed during recombinant production of *Um*AA10_cd that suggests modification of the yeast cell wall by LPMO.

### Conclusion

In the present study, we identified and characterized the catalytic domain of the first fungal LPMO belonging to the AA10 family. This LPMO, which is unique in the genome of *Ustilago maydis*, was found to be active on model chitin but also on chitin-containing *Um*FCW extracts. Importantly, we showed that the LPMO activity contributes to chitin hydrolysis by an endogenous GH18 chitinase (*Um*GH18A). We believe that the acquisition by the plant pathogen *U. maydis* of such unique oxidative activity, possibly by HGT, is not fortuitous and plays an important role in the fungus biology.

## MATERIALS AND METHODS

### Materials

Most chemicals, including Avicel and horseradish peroxidase (HRP), were purchased from Sigma-Aldrich. Chito-oligosaccharides were purchased from Megazyme (Wicklow, Ireland). Both α- and β-chitin were purchased from Mahtani Chitosan (Gujarat, India). PASC was prepared as described in reference (73).

### Bioinformatic analyses

To build up the phylogenetic tree, 197 AA10 amino acids’ sequences (catalytic domain only), including nine sequences from fungi (Ustilaginomycetes) and 46 sequences of characterized AA10 LPMOs, were selected. Multiple sequence alignment was performed using the MAFFT-L-INS-i methods via the MAFFT online platform (74). The phylogenetic tree was generated with IQ-TREE online platform (75), with the maximum likelihood method. The Whelan and Goldman amino acid substitution model was selected (76). Branch support was calculated by 1,000 Bootstrap repetitions (values displayed in percent on the tree). The tree was visualized in iTOL (77) and edited in Illustrator CC 2017. The C-terminal regions of unknown function were extracted and analyzed with the disorder predictor IUPred2A (46). The program returns a score between 0 and 1, which represents the probability for each residue of being part of a disordered region (score ≥ 0.5). *Um*AA10 C-terminal region was further analyzed with MobidB-lite 3.0 using default parameters and the AlphaFold pLDDT score [values ≤ 68.8; see reference (78)]. The properties of the dCTRs were derived from their primary sequence through the toolkit localCIDER (79).

### Cloning and production of recombinant enzymes

The proteins were produced using the in-house 3PE platform (*Pichia pastoris* protein express; www.platform3pe.com/) as described in reference (80). The nucleotide sequence coding for the AA10 of *Ustilago maydis* (Genbank ID XP_011391789.1) was codon optimized and synthesized (GenScript, Piscataway, USA) for expression in *Pichia pastoris* (syn. *Komagataella phaffii*). The region corresponding to the native signal sequence was kept, and upon production of the catalytic domain only (*Um*AA10_cd), the C-terminal extension region was removed (at position corresponding to between Gly203 and Gly204). The genes encoding *Serratia marcescens* GH20 (*Sm*GH20) (Genbank ID Q54468.1) and *U. maydis* GH18A (*Um*GH18A) (Genbank ID XP_011389118.1) were synthesized after codon optimization for *P. pastoris* (GenScript) using the full-length native sequence. Each gene was inserted into the expression vector pPICZαA (Invitrogen, Cergy-Pontoise, France) with a C-terminal (His)_6_-tag. PmeI-linearized pPICZαA recombinant plasmids were used to transform by electroporation competent *P. pastoris* cells (SuperMan5 for *Um*AA10_cd and *Sm*GH20, and X33 for *Um*GH18A). Zeocin-resistant transformants were then screened for protein production. The best-producing transformants were grown in 2 L of buffered glycerol-complex medium (BMGY) (10 g/L yeast extract, 20 g/L peptone, 3.4 g/L yeast nitrogen base without amino acids, 10 g/L ammonium sulfate, 10 g/L glycerol, potassium phosphate buffer pH 6.0, biotin 0.4 mg/L) in flasks at 30°C in an orbital shaker (200 rpm) for 16 h to an optical density ( OD_600_) of 2–6. Expression was induced by transferring cells into 400 mL of buffered methanol-complex medium (BMMY) (10 g/L yeast extract, 20 g/L peptone, 3.4 g/L yeast nitrogen base without amino acids, 10 g/L, ammonium sulfate, potassium phosphate buffer pH 6.0, biotin 0.4 mg/L). The medium was supplemented (initially and everyday) with 1% (vol/vol) methanol at 16°C for *Um*AA10_cd and 3% (vol/vol) methanol at 20°C for *Sm*GH20 and *Um*GH18A in an orbital shaker (200 rpm) for another 3 days. The cells were harvested by centrifugation (5,000 rpm, 10 min at 4°C), and just before purification, the pH of the supernatant was adjusted to 7.8 with sodium hydroxide and was filtered at 0.45 µm (Millipore, Burlington, Massachusetts, USA).

### Proteins purification

Filtered and pH-adjusted culture supernatants were loaded onto a 5 mL HisTrap HP column (GE Healthcare, Bus, France) equilibrated with buffer A (Tris-HCl 50 mM pH 7.8, NaCl 150 mM, imidazole 10 mM) that was connected to an Äkta purifier 100 (GE Healthcare). (His)_6_-tagged recombinant proteins were eluted with buffer B (Tris-HCl 50 mM pH 7.8, NaCl 150 mM, imidazole 500 mM). Fractions containing recombinant *Um*GH18A or *Sm*GH20 enzymes were pooled, concentrated, and dialyzed against Tris-HCl buffer 50 mM, pH 8.0. The concentrated sample of *Um*AA10_cd was further purified by size exclusion chromatography, using a HiLoad 16/600 Superdex 75 pg column (Cytivia, Illkirch, France) operated at 1 mL/min with sodium acetate buffer 50 mM, pH 5.2. The protein concentrations were determined by absorption at 280 nm using a Nanodrop ND-2000 spectrophotometer (Thermo Fisher Scientific, Illkirch, France) and extinction coefficient (*Sm*GH20: 152,555 M^−1^/cm; *Um*AA10_cd: 32,680 M^−1^/cm; and *Um*GH18A: 84,465 M^−1^/cm) was determined with ProtParam (Expasy). Protein purity was checked by analysis onto a 10% Tris-Glycine precast SDS-PAGE (BioRad, Gemenos, France), stained with Blue.

### ICP-MS analysis

Copper content was analyzed using ICP-MS as described in reference (81). The samples were mineralized, then diluted in ultrapure water, and analyzed by an ICAP Q apparatus (Thermo Electron, Les Ullis, France). The copper concentration was determined using Plasmalab (Thermo Electron) software, at *m*/*z* = 63 with an accuracy of ±5%.

### H_2_O_2_ consumption and production assay

The ability of *Um*AA10_cd to produce H_2_O_2_ (oxidase assay) was determined by absorbance measurement, using the Amplex red assay (Thermo Fisher Scientific) and HRP, as described by Kittl et al. (51). The reaction mixture (100 µL in 96-well microplate) contained sodium phosphate buffer (pH 7.0, 50 mM), HRP (0.1 mg/mL), Amplex red (100 µM), and *Um*AA10_cd (1 µM). The reaction, incubated at 23°C, was initiated by adding ascorbic acid (50 µM) and the release of resorufin was monitored at 575 nm during 40 min. Two control reactions were carried out in the same condition: one without *Um*AA10_cd and another without ascorbic acid. The stoichiometry of the resorufin release in regard to the H_2_O_2_ produced is 2:1. A standard curve of H_2_O_2_ was performed under the same conditions.

H_2_O_2_ consumption was measured using two different assays. The first one was carried out following the protocol described by Breslmayr et al. (58). This assay is based on the oxidation of 2,6-DMP to coerulignone by the enzyme in the presence of H_2_O_2_ (peroxidase reaction). The stoichiometry of the reaction is 2 H_2_O_2_ consumed or 2 molecules of 2,6-DMP oxidized per coerulignone produced. Reactions were run in a 100 µL mixture, containing borate buffer (50 mM, pH 7.5), *Um*AA10_cd (1 µM), 2,6-DMP (500 µM), and H_2_O_2_ (100 µM). The release of coerulignone was monitored at 469 nm in a plate reader (ε469 = 53,200 M^−1^/cm). The standard curve obtained was used to determine H_2_O_2_ consumption kinetic parameters. In the second reaction, H_2_O_2_ consumption was measured in the presence of the polysaccharide as described by Bissaro et al. (56), with a monitoring by the method described by Kittl et al. (51). Reactions were conducted in a 600 µL mixture containing sodium phosphate buffer (50 mM, pH 6.0), α-chitin as substrate (10 g/L), EDTA (50 µM), and *Um*AA10_cd (50 nM). After 10 min of incubation (30°C, 1,000 rpm), H_2_O_2_ (100 µM) was added to the mixture and the reaction was initiated by adding ascorbic acid (20 µM). Seventy-five microliters of the samples was taken from the mixture at 0, 5, 10, 15, 20, 30, and 60 min of reaction, filtered using a 96-well filtration plate (0.22 µm filters, Merck Millipore, Ireland and 25 µL of each filtered sample was added to 75 µL of oxidase reagent as described above. A H_2_O_2_ standard curve was realized in the condition of the oxidase assay.

### Enzymatic characterization of *Um*GH18A

Temperature and pH optima for *Um*GH18A were determined as described previously (82) with slight modifications. The optimal pH was determined using various buffers (50 mM), within the pH range of 3–9. The buffers used were sodium citrate (pH 3, 4, 5, and 6), sodium acetate (pH 4, 5, and 5.5), sodium phosphate (pH 6, 7, and 8), and Tris-HCl (pH 7.2, 8, and 9). While, for determining optimum temperature, the chitinase activity assay was performed under the optimum pH, within a temperature range of 20°C–70°C. In both experiments, the reaction mixture contained colloidal chitin (10 g/L) as the substrate and purified *Um*GH18A (1µM), incubated at the respective temperature under stirring at 800rpm for 1h. Chitinase activity was determined by analyzing the presence of reducing sugars using Schales’ assay as described previously (82).

Time course degradation of crystalline chitin substrates was performed by incubating *Um*GH18A (1 µM) with α- or β-chitin (10 g/L) under the optimal conditions and shaking at 1,000 rpm. Aliquots were collected at different time points between 1 and 24 h and filtered using a 96-well filter plate (0.45 µm filters; Merck Millipore, USA) operated by a Millipore vacuum manifold. The filtered samples were then mixed with an equal volume of 70% acetonitrile and analyzed using high-performance liquid chromatography (HPLC). The products obtained were separated on Shim-pack GIST NH2 column (5 µm, 4.6 × 250 mm, Shimadzu, Japan), through isocratic elution using 70% acetonitrile, with a 0.7 mL/min flow rate. Throughout the analysis, the column oven temperature was maintained at 45°C, and the products were detected at 210 nm. Quantification of the CHOS was performed essentially as described earlier (83).

### Enzymatic characterization of *Um*AA10_cd

To assess the enzymatic activity of *Um*AA10_cd, reactions were performed on model substrates (α- and β-chitin, Avicel, and PASC) and *Um*FCW extract. Activity on model chitin and *Um*FCW was carried out in 300 µL reaction mixture containing *Um*AA10_cd (1 µM), sodium phosphate buffer (50 mM, pH 6.0), and substrate (α- or β-chitin, or *Um*FCW; 10 g/L), which were incubated for 30 min (30°C, 1,000 rpm) before adding ascorbic acid (1 mM) and *Um*GH18A (0.1 µM). Reactions were incubated at 30°C (1,000 rpm) during 24 h and stopped by heat at 100°C during 10 min and then filtered at 0.22 µm using a 96-well filtration plate (0.22 µm filters, Merck Millipore, Ireland). To quantify released chito-oligosaccharides, *Sm*GH20 (1 µM) was added to the filtrate reaction and the reaction was run for 5 h (30°C, 1,000 rpm) and stopped by heat (100°C, 10 min). Standard C1-oxidized CHOS was prepared as described in reference (84). Activity tests on cellulose (Avicel or PASC) were carried out in 300 µL reaction mixture containing *Um*AA10_cd (1 µM), sodium phosphate buffer (50 mM, pH 6.0), and Avicel (10 g/L) or PASC (0.1%), which were incubated for 30 min (30°C, 1,000 rpm) before adding ascorbic acid (1 mM). Reactions were incubated for 24 h (30°C, 1,000 rpm), stopped by heat (100°C, 10 min), and then filtered using a 96-well filtration plate (0.22 µm filters, Merck Millipore, Ireland). All reactions were carried out in triplicate. Enzymatic reactions were diluted 10 times, and analyzed using a high-performance anion-exchange chromatography (HPAEC) coupled with pulsed amperometric detection (PAD) (Dionex ICS6000 system, Thermo Fisher Scientific, Waltham, MA, USA). The system is equipped with a CarboPac-PA1 guard column (2 × 50 mm) and a CarboPac-PA1 column (2 × 250 mm) kept at 30°C. Elution was carried out at a flow rate of 0.1 mL/min and 25 µL of the sample was injected. The solvents used were NaOH (100 mM; eluent A) and NaOAc (1 M) in NaOH (100 mM; eluent B). The column was preconditioned with 1.4% eluent B for 24 h, and then the following gradient was applied: 0–10 min, 1.4% B; 10–32 min, 1.4%–14% B; 32–46 min, 1.4% B. Integration was performed using the Chromeleon 7.2.10 chromatography data software.

### *Ustilago maydis* growth conditions and cell wall preparation

The *U. maydis* strain [521/FGSC 9021; see reference (33)], which was provided by the CIRM-CF collection (strain CIRM-BRFM1093) (85) was grown in 100 mL of yeast extract peptone dextose (YPD) medium (10 g/L yeast extract, 20 g/L peptone, 20 g/L dextrose) for 48 h at 28°C in 250-mL baffled Erlenmeyer flasks under orbital agitation (150 rpm). Cells were then harvested, washed once in H_2_O by centrifugation (1,500*g*, 10 min), and stored at −80°C in 20% glycerol at 10^7^ cells/mL. To produce material for sequential extraction (Fig. S9), eight Roux flasks containing 180 mL of YPD medium were inoculated at 10^5^ cells/mL and incubated for 17 days at 28°C. The resulting mycelium was then harvested and washed three times with H_2_O by filtration on Miracloth and lyophilized. Five grams of this material was resuspended in 500 mL H_2_O, homogenized using Ultra-Turrax (13,500 rpm, 2 min), and boiled for 3 h. After centrifugation (6,000*g*, 10 min), the supernatant was discarded and the pellet was resuspended in 500 mL of NaOH (1.25 M) for 4 h at 60°C. After another centrifugation step, the alkali insoluble residue was washed three times using centrifugation (8,000*g*, 20 min) in 1 L of H_2_O. The pellet referred to as *Um*FCW, which contains chitin, was lyophilized, weighted (967 mg), resuspended in H_2_O in 1:1 ratio (wt/vol), and stored at 4°C until further use.

### Compositional analysis of fungal cell wall polysaccharides

Identification and quantification of polysaccharide neutral sugars were performed by gas-liquid chromatography (GC) after sulfuric acid degradation as described in reference (86). Briefly, 5 mg of dried alkali insoluble fraction was dispersed in 2 N trifluoroacetic acid and then hydrolyzed 90 min at 121°C. Neutral monosaccharides were converted to alditol acetates and analyzed on a TG-225 GC Column (30 × 0.32 mm ID) using TRACE Ultra Gas Chromatograph (Thermo Scientific; temperature 205°C, carrier gas H_2_). Standard sugars’ solution and inositol as internal standard were used for calibration.

Glucosamine residues were quantified after acid hydrolysis [adapted from reference (87)] and HPAEC-PAD analyses [adapted from reference (88)]. Briefly, 5 mg of dried alkali insoluble fraction was dispersed in acetic acid 1% and hydrolyzed in concentrated HCl 10 M at 105°C during 6 h. After dilution in milli-Q water, glucosamine residues were quantified by HPAEC-PAD (ICS-6000, Thermo Scientific) using a CarboPac PA20 column (2 × 250 mm, Thermo Scientific), thermostated at 30°C. An isocratic elution of 1.7 mM sodium acetate (NaOAc) in 1 mM NaOH was used at a 0.25 mL/min flow rate. Standard glucosamine solutions were used for calibration.

### Labeling of fungal cell wall chitin

Fungal cell wall chitin was labeled using the lectin WGA-AF488 (wheat germ agglutinin conjugated to Alexa Fluor 488; Thermo Fisher Scientific). The *U. maydis* mycelium (~ 1 cm²) was disrupted in 1 mL of H_2_O using a FastPrep homogenizer (MP Biomedicals, Illkirch-Graffenstaden, France). Fifty microliters of *U. maydis* mycelium, α-chitin, or *Um*FCW-AI (all at 20 g/L) was diluted 10-fold in phosphate-buffered saline (PBS) solution with bovine serum albumin (BSA) 0.1% (wt/vol) with or without WGA-AF488 at 1 µg/mL. After 1 h of incubation, samples were washed three times in PBS-BSA 0.1% and resuspended in 500 µL of PBS before imaging using an Olympus microscope BH2 with fluorescence (Rungis, France) at 500× magnification. Images were captured during 1 s using the Archimed software (v5.6.0, Microvision Instruments, Evry, France).

## SUPPLEMENTAL MATERIAL

Supplemental figures aem.00573-23-s0001.docx Figures S1 to S9 and additional references.

ASM does not own the copyrights to Supplemental Material that may be linked to, or accessed through, an article. The authors have granted ASM a non-exclusive, world-wide license to publish the Supplemental Material files. Please contact the corresponding author directly for reuse.

## References

[1] Vaaje-Kolstad G, Westereng B, Horn SJ, Liu Z, Zhai H, Sørlie M, Eijsink VGH. 2010. An oxidative enzyme boosting the enzymatic conversion of recalcitrant polysaccharides. Science 330:219–222. doi:10.1126/science.1192231 20929773

[2] Phillips CM, Beeson WTI, Cate JH, Marletta MA. 2011. Cellobiose dehydrogenase and a copper-dependent polysaccharide monooxygenase potentiate cellulose degradation by Neurospora crassa. ACS Chem Biol 6:1399–1406. doi:10.1021/cb200351y 22004347

[3] Forsberg Z, Vaaje-Kolstad G, Westereng B, Bunæs AC, Stenstrøm Y, MacKenzie A, Sørlie M, Horn SJ, Eijsink VGH. 2011. Cleavage of cellulose by a CBM33 protein. Protein Sci 20:1479–1483. doi:10.1002/pro.689 21748815 PMC3190143

[4] Bissaro B, Várnai A, Røhr ÅK, Eijsink VGH. 2018. Oxidoreductases and reactive oxygen species in conversion of lignocellulosic biomass. Microbiol Mol Biol Rev 82:e00029-18. doi:10.1128/MMBR.00029-18 30257993 PMC6298611

[5] Eibinger M, Ganner T, Bubner P, Rošker S, Kracher D, Haltrich D, Ludwig R, Plank H, Nidetzky B. 2014. Cellulose surface degradation by a Lytic polysaccharide monooxygenase and its effect on cellulase hydrolytic efficiency. J Biol Chem 289:35929–35938. doi:10.1074/jbc.M114.602227 25361767 PMC4276861

[6] Villares A, Moreau C, Bennati-Granier C, Garajova S, Foucat L, Falourd X, Saake B, Berrin J-G, Cathala B. 2017. Lytic polysaccharide monooxygenases disrupt the cellulose fibers structure. Sci Rep 7:40262. doi:10.1038/srep40262 28071716 PMC5223172

[7] Tokin R, Ipsen JØ, Westh P, Johansen KS. 2020. The synergy between LPMOs and cellulases in enzymatic saccharification of cellulose is both enzyme- and substrate-dependent. Biotechnol Lett 42:1975–1984. doi:10.1007/s10529-020-02922-0 32458293

[8] Johansen KS. 2016. Lytic polysaccharide monooxygenases: the microbial power tool for lignocellulose degradation. Trends Plant Sci 21:926–936. doi:10.1016/j.tplants.2016.07.012 27527668

[9] Chylenski P, Bissaro B, Sørlie M, Røhr ÅK, Várnai A, Horn SJ, Eijsink VGH. 2019. Lytic polysaccharide monooxygenases in enzymatic processing of lignocellulosic biomass. ACS Catal 9:4970–4991. doi:10.1021/acscatal.9b00246

[10] Wang D, Li J, Salazar-Alvarez G, McKee LS, Srivastava V, Sellberg JA, Bulone V, Hsieh YSY. 2018. Production of functionalised chitins assisted by fungal lytic polysaccharide monooxygenase. Green Chem 20:2091–2100. doi:10.1039/C8GC00422F

[11] Westereng B, Kračun SK, Leivers S, Arntzen MØ, Aachmann FL, Eijsink VGH. 2020. Synthesis of glycoconjugates utilizing the regioselectivity of a lytic polysaccharide monooxygenase. Sci Rep 10:13197. doi:10.1038/s41598-020-69951-7 32764705 PMC7411024

[12] Moreau C, Tapin-Lingua S, Grisel S, Gimbert I, Le Gall S, Meyer V, Petit-Conil M, Berrin J-G, Cathala B, Villares A. 2019. Lytic polysaccharide monooxygenases (LPMOS) facilitate cellulose nanofibrils production. Biotechnol Biofuels 12:156. doi:10.1186/s13068-019-1501-0 31249619 PMC6589874

[13] Vandhana TM, Reyre J-L, Sushmaa D, Berrin J-G, Bissaro B, Madhuprakash J. 2022. On the expansion of biological functions of lytic polysaccharide monooxygenases. New Phytol 233:2380–2396. doi:10.1111/nph.17921 34918344

[14] Drula E, Garron M-L, Dogan S, Lombard V, Henrissat B, Terrapon N. 2022. The carbohydrate-active enzyme database: functions and literature. Nucleic Acids Res 50:D571–D577. doi:10.1093/nar/gkab1045 34850161 PMC8728194

[15] Vaaje-Kolstad G, Houston DR, Riemen AHK, Eijsink VGH, van Aalten DMF. 2005. Crystal structure and binding properties of the Serratia marcescens chitin-binding protein CBP21. J Biol Chem 280:11313–11319. doi:10.1074/jbc.M407175200 15590674

[16] Forsberg Z, Mackenzie AK, Sørlie M, Røhr ÅK, Helland R, Arvai AS, Vaaje-Kolstad G, Eijsink VGH. 2014. Structural and functional characterization of a conserved pair of bacterial cellulose-oxidizing lytic polysaccharide monooxygenases. Proc Natl Acad Sci U S A 111:8446–8451. doi:10.1073/pnas.1402771111 24912171 PMC4060697

[17] Loose JSM, Forsberg Z, Fraaije MW, Eijsink VGH, Vaaje-Kolstad G. 2014. A rapid quantitative activity assay shows that the Vibrio cholerae Colonization factor Gbpa is an active Lytic polysaccharide Monooxygenase. FEBS Lett 588:3435–3440. doi:10.1016/j.febslet.2014.07.036 25109775

[18] Paspaliari DK, Loose JSM, Larsen MH, Vaaje-Kolstad G. 2015. Listeria monocytogenes has a functional Chitinolytic system and an active Lytic polysaccharide Monooxygenase. FEBS J 282:921–936. doi:10.1111/febs.13191 25565565

[19] Zhang H, Zhao Y, Cao H, Mou G, Yin H. 2015. Expression and characterization of a lytic polysaccharide monooxygenase from Bacillus thuringiensis. Int J Biol Macromol 79:72–75. doi:10.1016/j.ijbiomac.2015.04.054 25936286

[20] Chaplin AK, Wilson MT, Hough MA, Svistunenko DA, Hemsworth GR, Walton PH, Vijgenboom E, Worrall JAR. 2016. Heterogeneity in the histidine-brace copper coordination sphere in auxiliary activity family 10 (AA10) lytic polysaccharide monooxygenases. J Biol Chem 291:12838–12850. doi:10.1074/jbc.M116.722447 27129229 PMC4933455

[21] Fowler CA, Sabbadin F, Ciano L, Hemsworth GR, Elias L, Bruce N, McQueen-Mason S, Davies GJ, Walton PH. 2019. Discovery, activity and Characterisation of an Aa10 Lytic polysaccharide Oxygenase from the Shipworm Symbiont Teredinibacter Turnerae. Biotechnol Biofuels 12:232. doi:10.1186/s13068-019-1573-x 31583018 PMC6767633

[22] Guo X, An Y, Jiang L, Zhang J, Lu F, Liu F. 2022. The discovery and enzymatic characterization of a novel AA10 LPMO from Bacillus amyloliquefaciens with dual substrate specificity. Int J Biol Macromol 203:457–465. doi:10.1016/j.ijbiomac.2022.01.110 35065137

[23] Skåne A, Edvardsen PK, Cordara G, Loose JSM, Leitl KD, Krengel U, Sørum H, Askarian F, Vaaje-Kolstad G. 2022. Chitinolytic enzymes contribute to the pathogenicity of Aliivibrio salmonicida LFI1238 in the invasive phase of cold-water Vibriosis. BMC Microbiol 22:194. doi:10.1186/s12866-022-02590-2 35941540 PMC9361615

[24] Chiu E, Hijnen M, Bunker RD, Boudes M, Rajendran C, Aizel K, Oliéric V, Schulze-Briese C, Mitsuhashi W, Young V, Ward VK, Bergoin M, Metcalf P, Coulibaly F. 2015. Structural basis for the enhancement of virulence by viral spindles and their in vivo crystallization. Proc Natl Acad Sci U S A 112:3973–3978. doi:10.1073/pnas.1418798112 25787255 PMC4386404

[25] Li F, Liu Y, Liu Y, Li Y, Yu H. 2022. Heterologous expression and characterization of a novel lytic polysaccharide monooxygenase from Natrialbaceae archaeon and its application for chitin biodegradation. Bioresour Technol 354:127174. doi:10.1016/j.biortech.2022.127174 35436543

[26] Li F-W, Brouwer P, Carretero-Paulet L, Cheng S, de Vries J, Delaux P-M, Eily A, Koppers N, Kuo L-Y, Li Z, Simenc M, Small I, Wafula E, Angarita S, Barker MS, Bräutigam A, dePamphilis C, Gould S, Hosmani PS, Huang Y-M, Huettel B, Kato Y, Liu X, Maere S, McDowell R, Mueller LA, Nierop KGJ, Rensing SA, Robison T, Rothfels CJ, Sigel EM, Song Y, Timilsena PR, Van de Peer Y, Wang H, Wilhelmsson PKI, Wolf PG, Xu X, Der JP, Schluepmann H, Wong G-S, Pryer KM. 2018. Fern genomes elucidate land plant evolution and cyanobacterial symbioses. 7. Nat Plants 4:460–472. doi:10.1038/s41477-018-0188-8 29967517 PMC6786969

[27] Yadav SK, Archana, Singh R, Singh PK, Vasudev PG. 2019. Insecticidal fern protein Tma12 is possibly a lytic polysaccharide monooxygenase. Planta 249:1987–1996. doi:10.1007/s00425-019-03135-0 30903269

[28] Jiang W-X, Li P-Y, Chen X-L, Zhang Y-S, Wang J-P, Wang Y-J, Sheng Q, Sun Z-Z, Qin Q-L, Ren X-B, Wang P, Song X-Y, Chen Y, Zhang Y-Z. 2022. A pathway for chitin oxidation in marine bacteria. Nat Commun 13:5899. doi:10.1038/s41467-022-33566-5 36202810 PMC9537276

[29] Wong E, Vaaje-Kolstad G, Ghosh A, Hurtado-Guerrero R, Konarev PV, Ibrahim AFM, Svergun DI, Eijsink VGH, Chatterjee NS, van Aalten DMF, Ghosh P. 2012. The Vibrio cholerae colonization factor GbpA possesses a modular structure that governs binding to different host surfaces. PLoS Pathog 8:e1002373. doi:10.1371/journal.ppat.1002373 22253590 PMC3257281

[30] Garcia-Gonzalez E, Poppinga L, Fünfhaus A, Hertlein G, Hedtke K, Jakubowska A, Genersch E. 2014. Paenibacillus larvae chitin-degrading protein PlCBP49 is a key virulence factor in american foulbrood of honey bees. PLoS Pathog 10:e1004284. doi:10.1371/journal.ppat.1004284 25080221 PMC4117609

[31] Uchiyama S, Askarian F, Masson H, Sørensen HV, Zabihi MS, Golten O, Bunæs AC, Røhr ÅK, Kommedal E, Ludviksen JA, Arntzen MØ, Schmidt B, Zurich RH, van Sorge NM, Eijsink VGH, Krengel U, Mollnes TE, Lewis NE, Nizet V, Vaaje-Kolstad G, Mekasha S. 2021. The lytic polysaccharide monooxygenase CbpD promotes Pseudomonas aeruginosa virulence in systemic infection. 1. Nat Commun 12:1230. doi:10.1155/2021/5539595 33623002 PMC7902821

[32] Zhong X, Zhang L, van Wezel GP, Vijgenboom E, Claessen D, Palmer T, Pier GB. 2022. Role for a lytic polysaccharide monooxygenase in cell wall remodeling in Streptomyces coelicolor. mBio 13:e0045622. doi:10.1128/mbio.00456-22 35357207 PMC9040799

[33] Kämper J, Kahmann R, Bölker M, Ma L-J, Brefort T, Saville BJ, Banuett F, Kronstad JW, Gold SE, Müller O, Perlin MH, Wösten HAB, de Vries R, Ruiz-Herrera J, Reynaga-Peña CG, Snetselaar K, McCann M, Pérez-Martín J, Feldbrügge M, Basse CW, Steinberg G, Ibeas JI, Holloman W, Guzman P, Farman M, Stajich JE, Sentandreu R, González-Prieto JM, Kennell JC, Molina L, Schirawski J, Mendoza-Mendoza A, Greilinger D, Münch K, Rössel N, Scherer M, Vranes M, Ladendorf O, Vincon V, Fuchs U, Sandrock B, Meng S, Ho ECH, Cahill MJ, Boyce KJ, Klose J, Klosterman SJ, Deelstra HJ, Ortiz-Castellanos L, Li W, Sanchez-Alonso P, Schreier PH, Häuser-Hahn I, Vaupel M, Koopmann E, Friedrich G, Voss H, Schlüter T, Margolis J, Platt D, Swimmer C, Gnirke A, Chen F, Vysotskaia V, Mannhaupt G, Güldener U, Münsterkötter M, Haase D, Oesterheld M, Mewes H-W, Mauceli EW, DeCaprio D, Wade CM, Butler J, Young S, Jaffe DB, Calvo S, Nusbaum C, Galagan J, Birren BW. 2006. Insights from the genome of the biotrophic fungal plant pathogen Ustilago maydis. Nature 444:97–101. doi:10.1038/nature05248 17080091

[34] Brefort T, Doehlemann G, Mendoza-Mendoza A, Reissmann S, Djamei A, Kahmann R. 2009. Ustilago maydis as a pathogen. Annu Rev Phytopathol 47:423–445. doi:10.1146/annurev-phyto-080508-081923 19400641

[35] Geiser E, Reindl M, Blank LM, Feldbrügge M, Wierckx N, Schipper K. 2016. Activating intrinsic carbohydrate-active enzymes of the smut fungus Ustilago maydis for the degradation of plant cell wall components. Appl Environ Microbiol 82:5174–5185. doi:10.1128/AEM.00713-16 27316952 PMC4988183

[36] Couturier M, Navarro D, Olivé C, Chevret D, Haon M, Favel A, Lesage-Meessen L, Henrissat B, Coutinho PM, Berrin J-G. 2012. Post-Genomic analyses of fungal Lignocellulosic Biomass degradation reveal the unexpected potential of the plant pathogen Ustilago maydis. BMC Genomics 13:57. doi:10.1186/1471-2164-13-57 22300648 PMC3298532

[37] Reyre J-L, Grisel S, Haon M, Navarro D, Ropartz D, Le Gall S, Record E, Sciara G, Tranquet O, Berrin J-G, Bissaro B. 2022. The maize pathogen Ustilago maydis secretes glycoside hydrolases and carbohydrate oxidases directed toward components of the fungal cell wall. Appl Environ Microbiol 88:e0158122. doi:10.1128/aem.01581-22 36354345 PMC9746322

[38] Lanver D, Müller AN, Happel P, Schweizer G, Haas FB, Franitza M, Pellegrin C, Reissmann S, Altmüller J, Rensing SA, Kahmann R. 2018. The Biotrophic development of Ustilago maydis studied by RNA-seq analysis. Plant Cell 30:300–323. doi:10.1105/tpc.17.00764 29371439 PMC5868686

[39] Tamburrini KC, Terrapon N, Lombard V, Bissaro B, Longhi S, Berrin J-G. 2021. Bioinformatic analysis of lytic polysaccharide monooxygenases reveals the pan-families occurrence of intrinsically disordered C-terminal extensions. Biomolecules 11:1632. doi:10.3390/biom11111632 34827630 PMC8615602

[40] Jumper J, Evans R, Pritzel A, Green T, Figurnov M, Ronneberger O, Tunyasuvunakool K, Bates R, Žídek A, Potapenko A, Bridgland A, Meyer C, Kohl SAA, Ballard AJ, Cowie A, Romera-Paredes B, Nikolov S, Jain R, Adler J, Back T, Petersen S, Reiman D, Clancy E, Zielinski M, Steinegger M, Pacholska M, Berghammer T, Bodenstein S, Silver D, Vinyals O, Senior AW, Kavukcuoglu K, Kohli P, Hassabis D. 2021. Highly accurate protein structure prediction with AlphaFold. Nature 596:583–589. doi:10.1038/s41586-021-03819-2 34265844 PMC8371605

[41] Varadi M, Anyango S, Deshpande M, Nair S, Natassia C, Yordanova G, Yuan D, Stroe O, Wood G, Laydon A, Žídek A, Green T, Tunyasuvunakool K, Petersen S, Jumper J, Clancy E, Green R, Vora A, Lutfi M, Figurnov M, Cowie A, Hobbs N, Kohli P, Kleywegt G, Birney E, Hassabis D, Velankar S. 2022. Alphafold protein structure database: Massively expanding the structural coverage of protein-sequence space with high-accuracy models. Nucleic Acids Res. 50:D439–D444. doi:10.1093/nar/gkab1061 34791371 PMC8728224

[42] Aachmann FL, Sørlie M, Skjåk-Bræk G, Eijsink VGH, Vaaje-Kolstad G. 2012. NMR structure of a lytic polysaccharide monooxygenase provides insight into copper binding, protein dynamics, and substrate interactions. Proc. Natl. Acad. Sci. U.S.A 109:18779–18784. doi:10.1073/pnas.1208822109 23112164 PMC3503203

[43] Bissaro B, Streit B, Isaksen I, Eijsink VGH, Beckham GT, DuBois JL, Røhr ÅK. 2020. Molecular mechanism of the chitinolytic peroxygenase reaction. Proc. Natl. Acad. Sci. U.S.A 117:1504–1513. doi:10.1073/pnas.1904889117 31907317 PMC6983374

[44] Bissaro B, Isaksen I, Vaaje-Kolstad G, Eijsink VGH, Røhr ÅK. 2018. How a lytic polysaccharide monooxygenase binds crystalline chitin. Biochemistry 57:1893–1906. doi:10.1021/acs.biochem.8b00138 29498832

[45] Finn RD, Attwood TK, Babbitt PC, Bateman A, Bork P, Bridge AJ, Chang H-Y, Dosztányi Z, El-Gebali S, Fraser M, Gough J, Haft D, Holliday GL, Huang H, Huang X, Letunic I, Lopez R, Lu S, Marchler-Bauer A, Mi H, Mistry J, Natale DA, Necci M, Nuka G, Orengo CA, Park Y, Rawlings ND, Redaschi N, Pesseat S, Richardson L, Rivoire C, Sangrador-Vegas A, Sigrist C, Sillitoe I, Smithers B, Squizzato S, Piovesan D, Sutton G, Thanki N, Thomas PD, Tosatto SCE, Wu CH, Xenarios I, Yeh L-S, Young S-Y, Mitchell AL, Potter SC. 2017. Interpro in 2017—beyond protein family and domain annotations. Nucleic Acids Res 45:D190–D199. doi:10.1002/cpbi.40 27899635 PMC5210578

[46] Mészáros B, Erdos G, Dosztányi Z. 2018. IUPred2A: context-dependent prediction of protein disorder as a function of redox state and protein binding. Nucleic Acids Res 46:W329–W337. doi:10.1093/nar/gky384 29860432 PMC6030935

[47] Das RK, Pappu RV. 2013. Conformations of intrinsically disordered proteins are influenced by linear sequence distributions of oppositely charged residues. Proc Natl Acad Sci U S A 110:13392–13397. doi:10.1073/pnas.1304749110 23901099 PMC3746876

[48] Tedeschi G, Salladini E, Santambrogio C, Grandori R, Longhi S, Brocca S. 2018. Conformational response to charge clustering in synthetic intrinsically disordered proteins. Biochim Biophys Acta Gen Subj 1862:2204–2214. doi:10.1016/j.bbagen.2018.07.011 30025858

[49] Bianchi G, Longhi S, Grandori R, Brocca S. 2020. Relevance of electrostatic charges in compactness, aggregation, and phase separation of intrinsically disordered proteins. Int J Mol Sci 21:6208. doi:10.3390/ijms21176208 32867340 PMC7503639

[50] Votvik AK, Røhr ÅK, Bissaro B, Stepnov AA, Sørlie M, Eijsink VGH, Forsberg Z. 2023. Structural and functional characterization of the catalytic domain of a cell-wall anchored bacterial Lytic polysaccharide Monooxygenase from Streptomyces coelicolor. 1. Sci Rep 13:5345. doi:10.1038/s41598-023-32263-7 37005446 PMC10067821

[51] Kittl R, Kracher D, Burgstaller D, Haltrich D, Ludwig R. 2012. Production of four Neurospora crassa lytic polysaccharide monooxygenases in Pichia pastoris monitored by a fluorimetric assay. Biotechnol Biofuels 5:79. doi:10.1186/1754-6834-5-79 23102010 PMC3500269

[52] Quinlan RJ, Sweeney MD, Lo Leggio L, Otten H, Poulsen J-C, Johansen KS, Krogh K, Jørgensen CI, Tovborg M, Anthonsen A, Tryfona T, Walter CP, Dupree P, Xu F, Davies GJ, Walton PH. 2011. Insights into the oxidative degradation of cellulose by a copper metalloenzyme that exploits biomass components. Proc Natl Acad Sci U S A 108:15079–15084. doi:10.1073/pnas.1105776108 21876164 PMC3174640

[53] Keller MB, Badino SF, Blossom BM, McBrayer B, Borch K, Westh P. 2020. Promoting and impeding effects of lytic polysaccharide monooxygenases on glycoside hydrolase activity. ACS Sustainable Chem. Eng 8:14117–14126. doi:10.1021/acssuschemeng.0c04779

[54] Langner T, Öztürk M, Hartmann S, Cord-Landwehr S, Moerschbacher B, Walton JD, Göhre V. 2015. Chitinases are essential for cell separation in Ustilago maydis. Eukaryot Cell 14:846–857. doi:10.1128/EC.00022-15 25934689 PMC4551588

[55] Stock J, Sarkari P, Kreibich S, Brefort T, Feldbrügge M, Schipper K. 2012. Applying unconventional secretion of the endochitinase Cts1 to export heterologous proteins in Ustilago maydis. J Biotechnol 161:80–91. doi:10.1016/j.jbiotec.2012.03.004 22446315

[56] Bissaro B, Røhr ÅK, Müller G, Chylenski P, Skaugen M, Forsberg Z, Horn SJ, Vaaje-Kolstad G, Eijsink VGH. 2017. Oxidative cleavage of polysaccharides by monocopper enzymes depends on H_2_O_2_. Nat Chem Biol 13:1123–1128. doi:10.1038/nchembio.2470 28846668

[57] Bissaro B, Eijsink VGH. 2023. Lytic polysaccharide monooxygenases: enzymes for controlled and site-specific fenton-like chemistry. Essays Biochem 67:575–584. doi:10.1042/EBC20220250 36734231 PMC10154617

[58] Breslmayr E, Hanžek M, Hanrahan A, Leitner C, Kittl R, Šantek B, Oostenbrink C, Ludwig R. 2018. A fast and sensitive activity assay for lytic polysaccharide monooxygenase. Biotechnol Biofuels 11:79. doi:10.1186/s13068-018-1063-6 29588664 PMC5865291

[59] Hangasky JA, Iavarone AT, Marletta MA. 2018. Reactivity of O_2_ versus H_2_O_2_ with polysaccharide monooxygenases. Proceedings of the National Academy of Sciences 115:4915–4920. doi:10.1073/pnas.1801153115 PMC5949000 29686097

[60] Torbjörnsson M, Hagemann MM, Ryde U, Hedegård ED. 2023. Histidine oxidation in lytic polysaccharide monooxygenase. J Biol Inorg Chem 28:317–328. doi:10.1007/s00775-023-01993-4 36828975 PMC10036459

[61] Fonseca FL, Guimarães AJ, Kmetzsch L, Dutra FF, Silva FD, Taborda CP, Araujo G de S, Frases S, Staats CC, Bozza MT, Schrank A, Vainstein MH, Nimrichter L, Casadevall A, Rodrigues ML. 2013. Binding of the wheat germ lectin to Cryptococcus neoformans chitooligomers affects multiple mechanisms required for fungal pathogenesis. Fungal Genet Biol 60:64–73. doi:10.1016/j.fgb.2013.04.005 23608320 PMC4294701

[62] Metcalf JA, Funkhouser-Jones LJ, Brileya K, Reysenbach A-L, Bordenstein SR. 2014. Antibacterial gene transfer across the tree of life. Elife 3:e04266. doi:10.7554/eLife.04266 25422936 PMC4241558

[63] Shin NR, Doucet D, Pauchet Y. 2022. Duplication of horizontally acquired GH5_2 enzymes played a central role in the evolution of longhorned beetles. Mol Biol Evol 39:msac128. doi:10.1093/molbev/msac128 35763818 PMC9246334

[64] Husnik F, McCutcheon JP. 2018. Functional horizontal gene transfer from bacteria to eukaryotes. Nat Rev Microbiol 16:67–79. doi:10.1038/nrmicro.2017.137 29176581

[65] Gow NAR, Latge J-P, Munro CA. 2017. The fungal cell wall: structure, biosynthesis, and function. Microbiol Spectr 5. doi:10.1128/microbiolspec.FUNK-0035-2016 PMC11687499 28513415

[66] Davì V, Tanimoto H, Ershov D, Haupt A, De Belly H, Le Borgne R, Couturier E, Boudaoud A, Minc N. 2018. Mechanosensation dynamically coordinates polar growth and cell wall assembly to promote cell survival. Dev Cell 45:170–182. doi:10.1016/j.devcel.2018.03.022 29689193

[67] Ene IV, Adya AK, Wehmeier S, Brand AC, MacCallum DM, Gow NAR, Brown AJP. 2012. Host carbon sources modulate cell wall architecture, drug resistance and virulence in a fungal pathogen. Cell Microbiol 14:1319–1335. doi:10.1111/j.1462-5822.2012.01813.x 22587014 PMC3465787

[68] Chakraborty A, Fernando LD, Fang W, Dickwella Widanage MC, Wei P, Jin C, Fontaine T, Latgé J-P, Wang T. 2021. A molecular vision of fungal cell wall organization by functional genomics and solid-state NMR. 1. Nat Commun 12:6346. doi:10.1038/s41467-021-26749-z 34732740 PMC8566572

[69] Rizzi YS, Happel P, Lenz S, Urs MJ, Bonin M, Cord-Landwehr S, Singh R, Moerschbacher BM, Kahmann R. 2021. Chitosan and chitin deacetylase activity are necessary for development and virulence of Ustilago maydis. mBio 12:e03419-20. doi:10.1128/mBio.03419-20 33653886 PMC8092297

[70] Detomasi TC, Rico-Ramírez AM, Sayler RI, Gonçalves AP, Marletta MA, Glass NL. 2022. A moonlighting function of a chitin polysaccharide monooxygenase, CWR-1, in Neurospora crassa allorecognition. Elife 11:e80459. doi:10.7554/eLife.80459 36040303 PMC9550227

[71] Probst C, Hallas-Møller M, Ipsen JØ, Brooks JT, Andersen K, Haon M, Berrin J-G, Martens HJ, Nichols CB, Johansen KS, Alspaugh JA. 2023. A fungal Lytic polysaccharide Monooxygenase is required for cell wall integrity, thermotolerance, and virulence of the fungal human pathogen Cryptococcus neoformans. PLOS pathogens 19:e1010946. doi:10.1371/journal.ppat.1010946 37099613 PMC10166503

[72] Fernando LD, Dickwella Widanage MC, Penfield J, Lipton AS, Washton N, Latgé J-P, Wang P, Zhang L, Wang T. 2021. Structural polymorphism of chitin and chitosan in fungal cell walls from solid-state NMR and principal component analysis. Front Mol Biosci 8:727053. doi:10.3389/fmolb.2021.727053 34513930 PMC8423923

[73] Wood TM. 1988. Preparation of crystalline, amorphous, and dyed cellulase substrates, p 19–25. In In methods in Enzymology. Academic Press.

[74] Katoh K, Rozewicki J, Yamada KD. 2019. MAFFT online service: multiple sequence alignment, interactive sequence choice and visualization. Brief Bioinform 20:1160–1166. doi:10.1093/bib/bbx108 28968734 PMC6781576

[75] Nguyen L-T, Schmidt HA, von Haeseler A, Minh BQ. 2015. IQ-TREE: a fast and effective stochastic algorithm for estimating maximum-likelihood phylogenies. Mol Biol Evol 32:268–274. doi:10.1093/molbev/msu300 25371430 PMC4271533

[76] Whelan S, Goldman N. 2001. A general empirical model of protein evolution derived from multiple protein families using a maximum-likelihood approach. Mol Biol Evol 18:691–699. doi:10.1093/oxfordjournals.molbev.a003851 11319253

[77] Letunic I, Bork P. 2021. Interactive tree of life (iTOL) V5: an online tool for phylogenetic tree display and annotation. Nucleic Acids Res 49:W293–W296. doi:10.1093/nar/gkab301 33885785 PMC8265157

[78] Piovesan D, Monzon AM, Tosatto SCE. 2022. Intrinsic protein disorder and conditional folding in AlphaFoldDB. Protein Sci 31:e4466. doi:10.1002/pro.4466 36210722 PMC9601767

[79] Holehouse AS, Das RK, Ahad JN, Richardson MOG, Pappu RV. 2017. CIDER: resources to analyze sequence-ensemble relationships of intrinsically disordered proteins. Biophys J 112:16–21. doi:10.1016/j.bpj.2016.11.3200 28076807 PMC5232785

[80] Haon M, Grisel S, Navarro D, Gruet A, Berrin J-G, Bignon C. 2015. Recombinant protein production facility for fungal biomass-degrading enzymes using the yeast Pichia pastoris. Front Microbiol 6:1002. doi:10.3389/fmicb.2015.01002 26441929 PMC4585289

[81] Couturier M, Ladevèze S, Sulzenbacher G, Ciano L, Fanuel M, Moreau C, Villares A, Cathala B, Chaspoul F, Frandsen KE, Labourel A, Herpoël-Gimbert I, Grisel S, Haon M, Lenfant N, Rogniaux H, Ropartz D, Davies GJ, Rosso M-N, Walton PH, Henrissat B, Berrin J-G. 2018. Lytic xylan oxidases from wood-decay fungi unlock Biomass degradation. Nat Chem Biol 14:306–310. doi:10.1038/nchembio.2558 29377002

[82] Mukherjee S, Behera PK, Madhuprakash J. 2020. Efficient conversion of crystalline Chitin to N-acetylglucosamine and N,N'-diacetylchitobiose by the enzyme cocktail produced by Paenibacillus sp. LS1. Carbohydr Polym 250:116889. doi:10.1016/j.carbpol.2020.116889 33049827

[83] Madhuprakash J, Dalhus B, Rani TS, Podile AR, Eijsink VGH, Sørlie M. 2018. Key residues affecting transglycosylation activity in family 18 chitinases: insights into donor and acceptor subsites. Biochemistry 57:4325–4337. doi:10.1021/acs.biochem.8b00381 29939724

[84] Haddad Momeni M, Fredslund F, Bissaro B, Raji O, Vuong TV, Meier S, Nielsen TS, Lombard V, Guigliarelli B, Biaso F, Haon M, Grisel S, Henrissat B, Welner DH, Master ER, Berrin J-G, Abou Hachem M. 2021. Discovery of fungal oligosaccharide-oxidising flavo-enzymes with previously unknown substrates, redox-activity profiles and interplay with LPMOs. Nat Commun 12:2132. doi:10.1038/s41467-021-22372-0 33837197 PMC8035211

[85] Navarro D, Chaduli D, Taussac S, Lesage-Meessen L, Grisel S, Haon M, Callac P, Courtecuisse R, Decock C, Dupont J, Richard-Forget F, Fournier J, Guinberteau J, Lechat C, Moreau P-A, Pinson-Gadais L, Rivoire B, Sage L, Welti S, Rosso M-N, Berrin J-G, Bissaro B, Favel A. 2021. Large-scale phenotyping of 1,000 fungal strains for the degradation of non-natural, industrial compounds. Commun Biol 4:871. doi:10.1038/s42003-021-02401-w 34267314 PMC8282864

[86] Lahaye M, Falourd X, Laillet B, Le Gall S. 2020. Cellulose, pectin and water in cell walls determine apple flesh viscoelastic mechanical properties. Carbohydr Polym 232:115768. doi:10.1016/j.carbpol.2019.115768 31952582

[87] Yan X, Evenocheck HM. 2012. Chitosan analysis using acid hydrolysis and HPLC/UV. Carbohydrate Polymers 87:1774–1778. doi:10.1016/j.carbpol.2011.09.091

[88] Nagel A, Sirisakulwat S, Carle R, Neidhart S. 2014. An acetate-hydroxide gradient for the quantitation of the neutral sugar and uronic acid profile of pectins by HPAEC-PAD without postcolumn pH adjustment. J Agric Food Chem 62:2037–2048. doi:10.1021/jf404626d 24547908

