# Supplementary material for: The Ustilago maydis AA10 LPMO is active on fungal cell wall chitin
Source: Appl Environ Microbiol. 2023 Sep 13;89(10):e00573-23. doi: 10.1128/aem.00573-23 (PMC10617569; doi:10.1128/aem.00573-23)
Supplement: Supplemental figures — Figures S1 to S9 and additional references. [file aem.00573-23-s0001.docx]

SUPPORTING INFORMATION FOR

**The *Ustilago maydis* AA10 LPMO is active on fungal cell wall chitin**

Roseline Assiah Yao^1^, Jean-Lou Reyre^1,2^, Ketty C. Tamburrini^1,3^, Mireille Haon^1,4^, Olivier Tranquet^1^, Akshay Nalubothula^5^, Saumashish Mukherjee^5^, Sophie Le Gall^6,7^, Sacha Grisel^1,4^, Sonia Longhi^3^, Jogi Madhuprakash^5^, Bastien Bissaro^1,^* and Jean-Guy Berrin^1,4,^*

^1^INRAE, Aix Marseille Univ., UMR 1163 Biodiversité et Biotechnologie Fongiques (BBF), Marseille, France

^2^IFP Energies Nouvelles, Rueil-Malmaison, France

^3^CNRS, Aix Marseille Univ., UMR 7257 Architecture et Fonction des Macromolécules Biologiques (AFMB), Marseille, France

^4^INRAE, Aix Marseille Univ., 3PE platform, Marseille, France

^5^Department of Plant Sciences, School of Life Sciences, University of Hyderabad, Gachibowli, Hyderabad, Telangana, India

^6^INRAE, UR1268 BIA, Nantes, France

^7^INRAE, PROBE research infrastructure, BIBS facility, Nantes, France

*corresponding authors:

Bastien Bissaro (bastien.bissaro@inrae.fr)

Jean-Guy Berrin (jean-guy.berrin@inrae.fr)

**Running title:** A fungal AA10 active on fungal cell wall

**This file includes:**

1. Supplementary Figures S1 to S9.
2. Additional References.


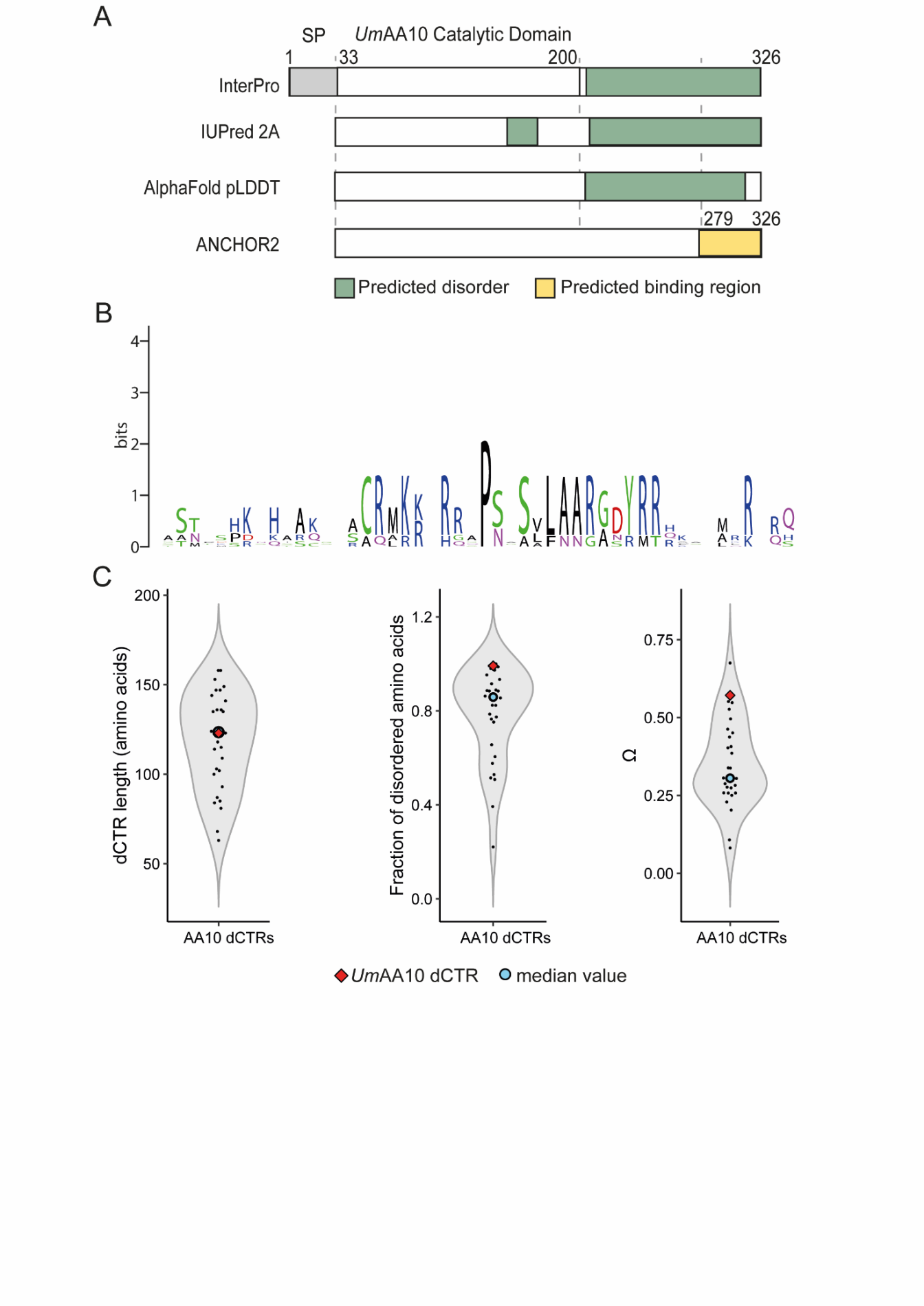


**Figure S1. Disorder prediction.** **(A)** Prediction of disorder and of disordered binding sites (DiBSs) as derived from Interpro, IUPred 2A (1), AlphaFold (2) and ANCHOR2 (1) Disordered regions are shown in green and disordered binding sites (DiBSs) in gold. **(B)** Amino-acid conservation of the last 50 residues across fungal AA10 LPMO-dCTRs visualized using WebLogo (<https://weblogo.berkeley.edu/logo.cgi> (3)). The x-axis indicates the position of the amino acid and the height of each letter represents the sequence conservation at the corresponding position. **(C)** On the left-hand side, violin plot showing the distribution of the length of dCTRs across AA10 LPMO dCTRs. In the middle, violin plot showing the fraction of disordered residues within dCTRs, as predicted by IUPred2A. On the right-hand side, violin plot showing the distribution of the patterning parameter Ω values of dCTRs (4). This parameter computes the distribution of Pro, Lys, Asp, Arg, Glu across the amino acid sequence: low Ω values indicate that these residues are well dispersed across the sequence, while high Ω values design their clustering. The blue dot in each violin plot represents the median length value.

**
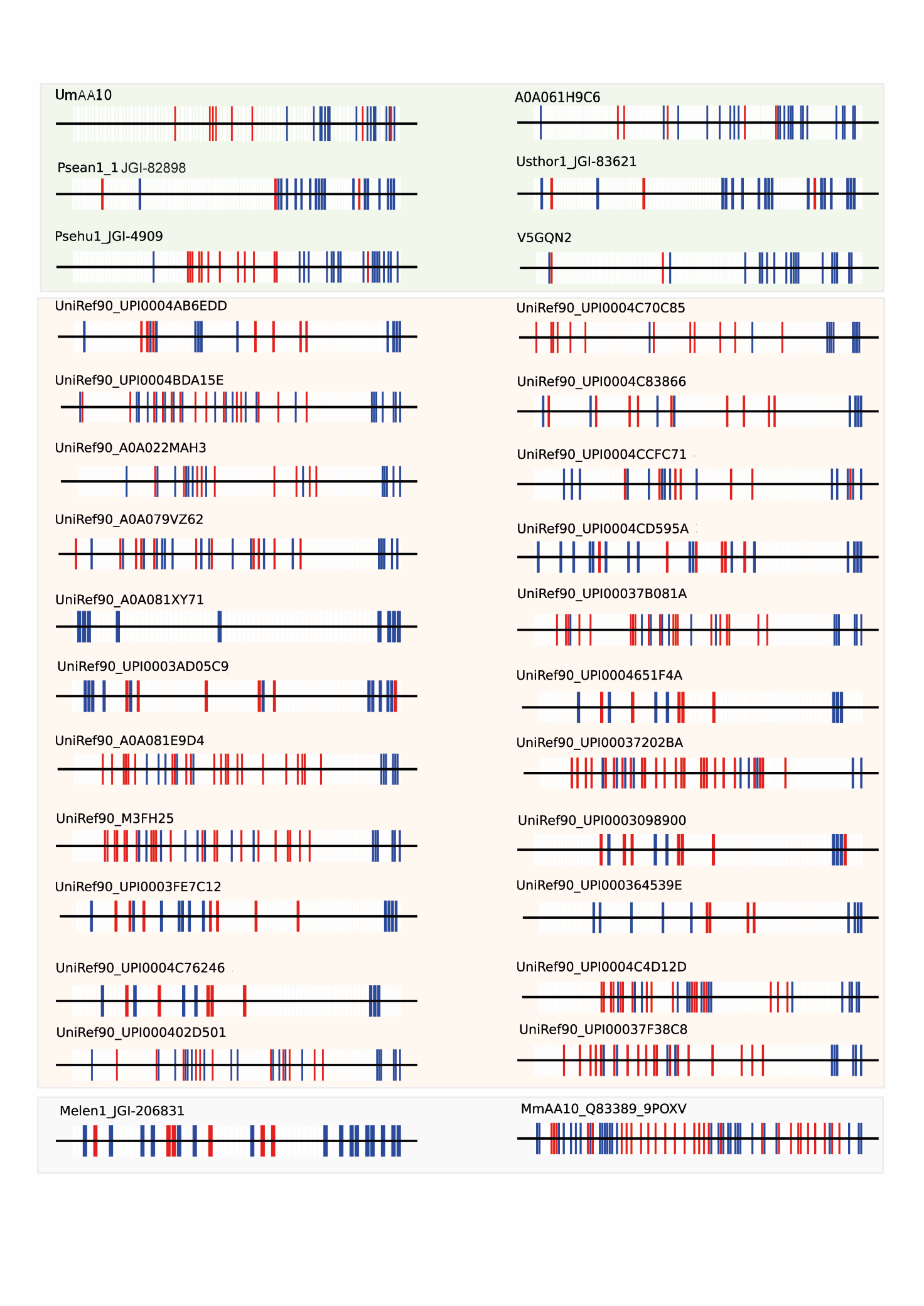
**

**Figure S2. Distribution of positive, negative and neutral residues in the dCTRs of AA10 LPMOs.** Asp and Glu residues are colored in red, Arg, Lys and His residues are colored in blue, and other residues are colored in white. Sequences shown with green and orange backgrounds correspond to dCTRs of fungal and bacterial AA10 LPMOs respectively, while the sequences shown with a grey background correspond to AA10 LPMOs from other organisms.


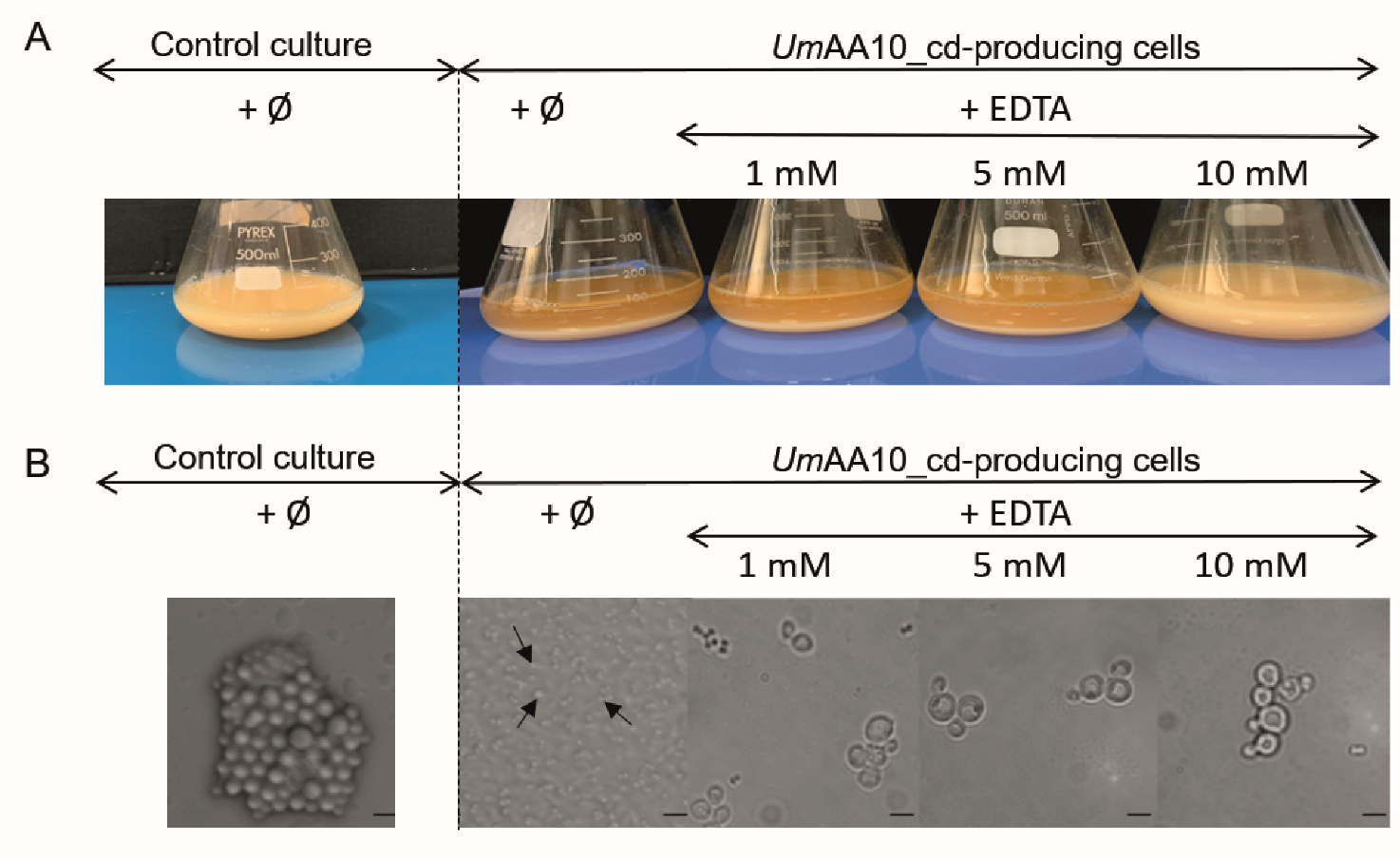


**Figure S3. *Um*AA10_cd production in the *Pichia pastoris* system**. **(A)** Cells sedimentation in culture medium at the end of the recombinant protein production (methanol media). From the left to the right: usual aspect of *P. pastoris* in culture medium (production of another recombinant protein) and *Um*AA10_cd-producing *P. pastoris* cells in similar culture conditions, where 0, 1, 5 or 10 mM of the copper-chelating agent EDTA was added. **(B)** *P. pastoris* cell morphology under “normal” conditions (left) and during *Um*AA10_cd production with increasing concentrations of EDTA. Black arrows show yeast cells. Scale bar: 20 µm.


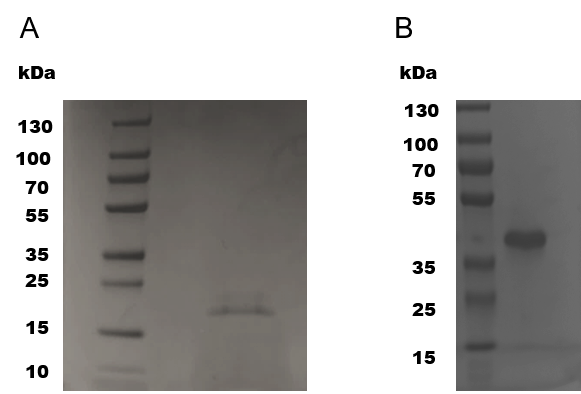


**Figure S4**. **SDS-PAGE analysis of (A) *Um*AA10_cd and (B) *Um*GH18A after purification.** The theoretical molecular mass of *Um*AA10_cd and *Um*GH18A are 19.5 kDa and 39.8 kDa, respectively.


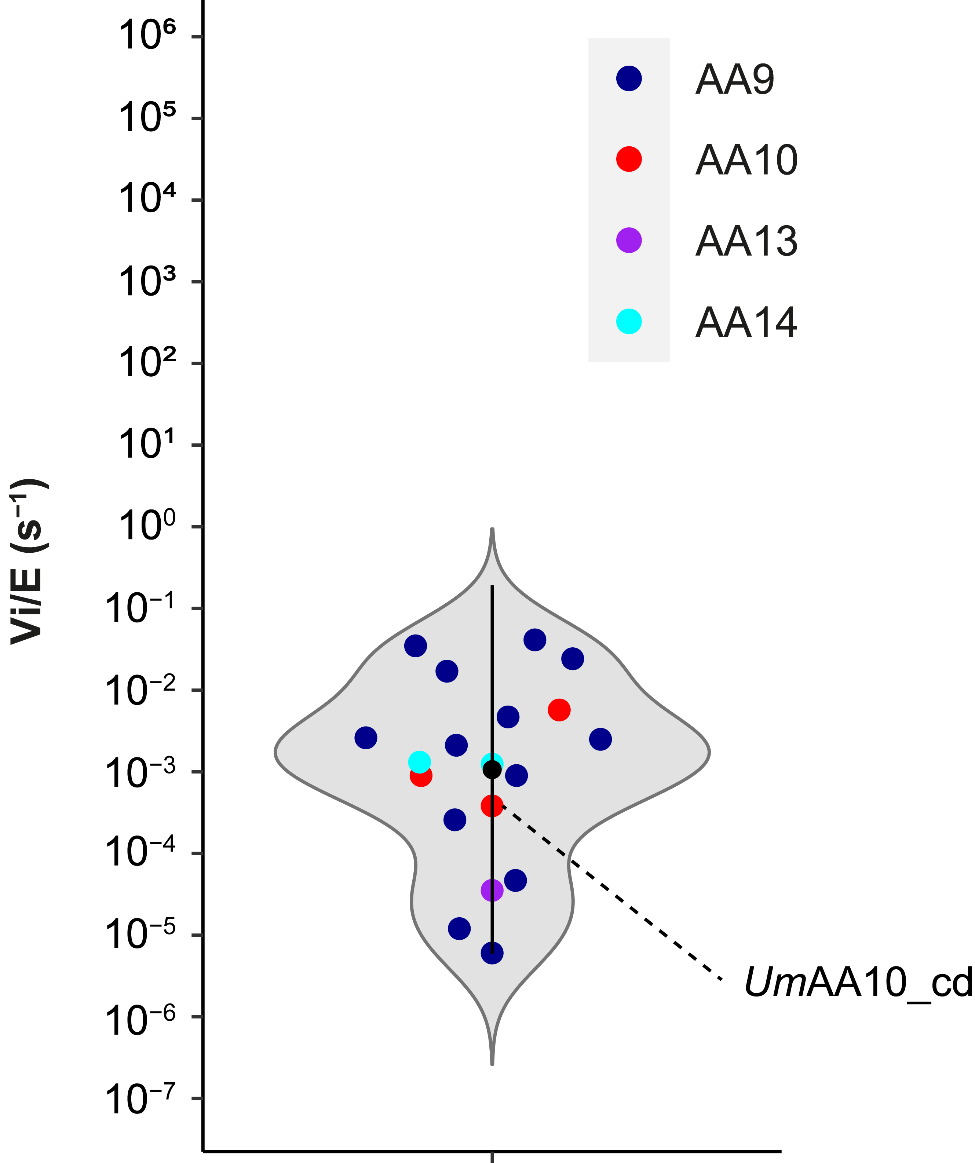


**Figure S5. Violin plot of oxidase rates of LPMOs.** The graph shows (log-scale) the H_2_O_2_ production rates (using mainly AscA as reductant) of 19 different LPMOs belonging to AA9, AA10, AA13 and AA14 families (the color code is provided in the figure). Data were taken from Bissaro et al. (5) and completed with the data obtained for *Um*AA10_cd in the present study. The black dot shows the mean value and the black line the double standard deviation.


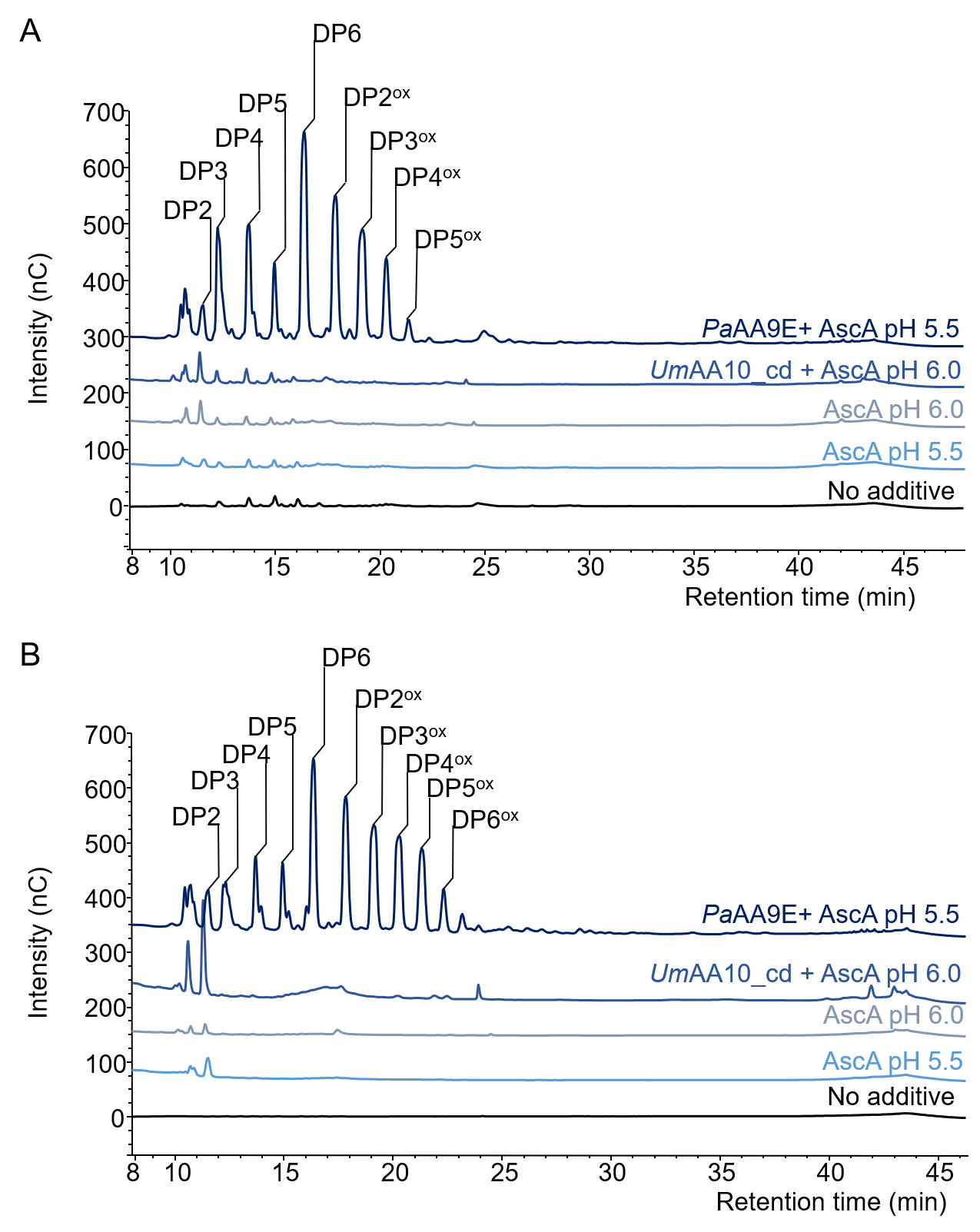


**Figure S6**. **Enzyme assays of *Um*AA10_cd on crystalline (A) and amorphous (B) cellulose.** The graphs show HPAEC-PAD chromatograms of products released from (A) Avicel (10 g.L^-1^) and (B) PASC (0.1%). Reactions were carried out under stirring (1000 rpm) at 30°C for 24 h in sodium acetate buffer (50 mM, pH 5.5) for those containing *Pa*AA9E (1 µM) or in sodium phosphate buffer (50 mM, pH 6.0) for those containing *Um*AA10_cd (1 µM). The *Pa*AA9E LPMO from *Podospora anserina* (6) was used as a control enzyme. Main products are cello-oligosaccharides of degree of polymerization (DP) from 2 to 6, either non-modified (DP1 to DP6) or C1-oxidized (DP1^ox^-DP6^ox^).


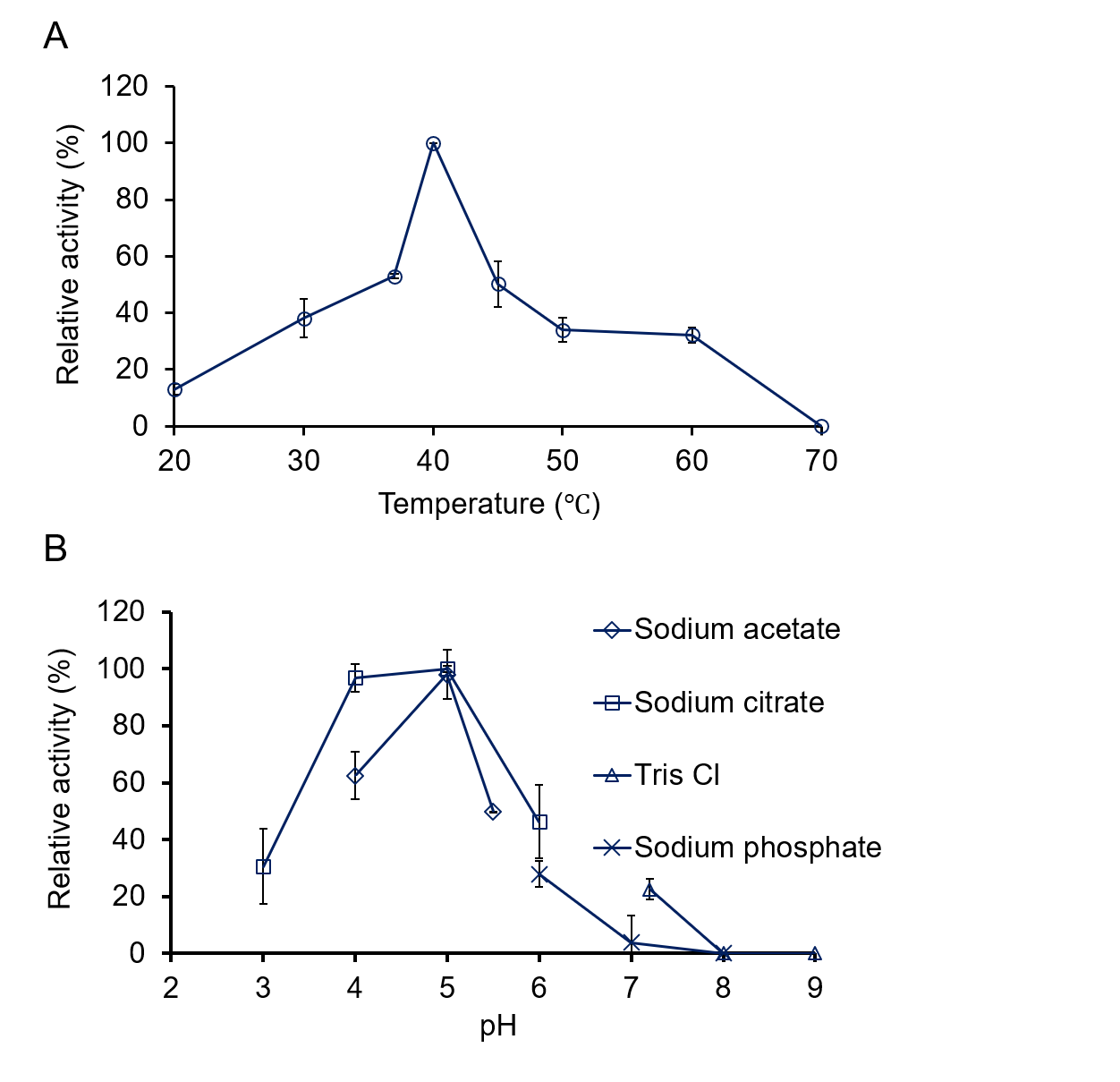


**Figure S7. Determination of optimum temperature and pH of *Um*GH18A.** (A) Optimum temperature for *Um*GH18A was determined by incubating *Um*GH18A (1 μM) with 10 g.L^-1^ colloidal chitin at various temperatures (20–70°C) for 1 h, and the relative activities were plotted against the temperature. (B) The optimum pH was measured in different buffers (50 mM strength) at various pH values. The enzyme load, substrate concentration and time of incubation were same as the temperature optima experiment and the relative activities were plotted against the pH. Data points show average values and the error bars indicate standard deviations from three independent replicates.

**
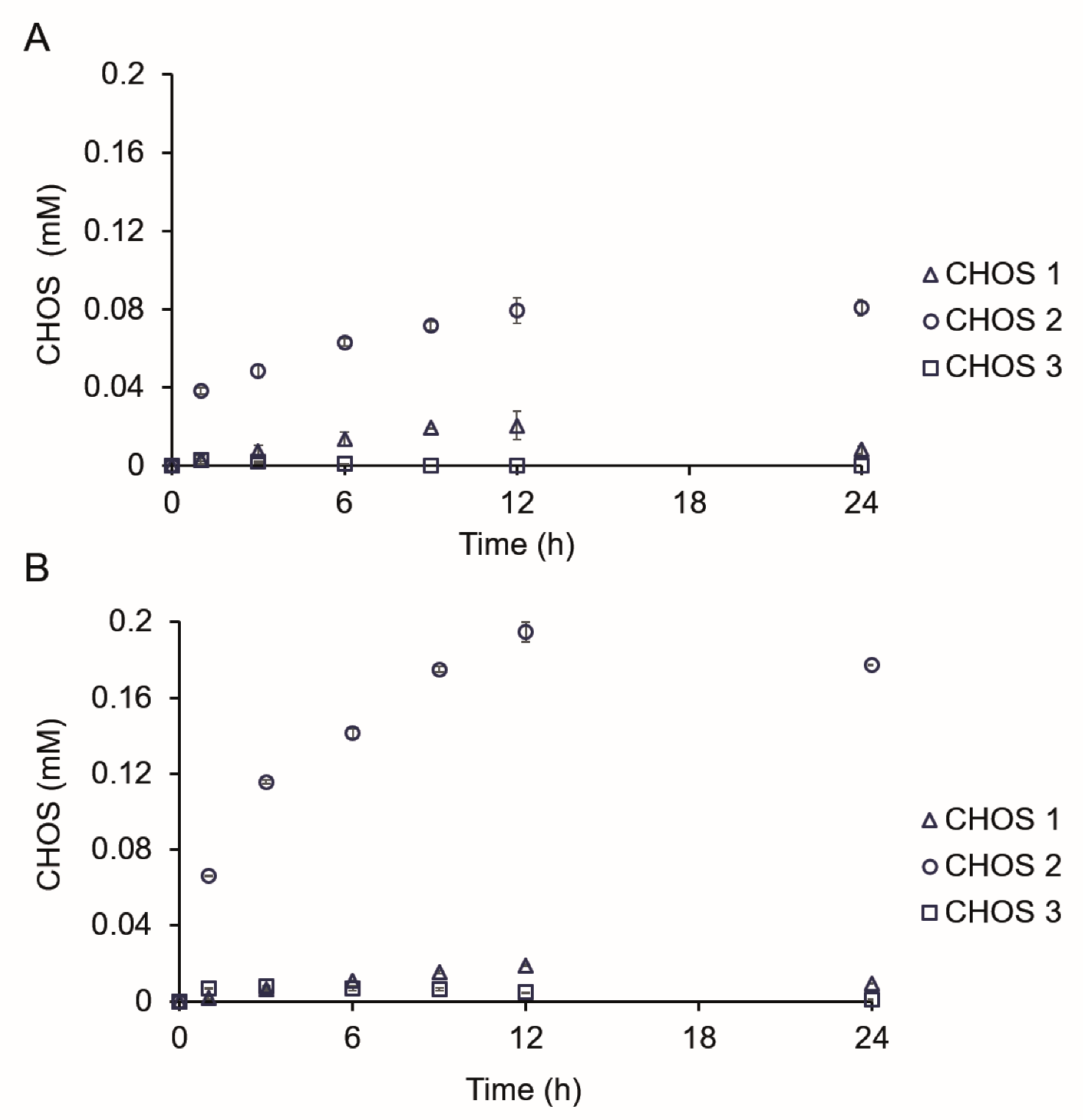
Figure S8. Time-course analysis of the chito-oligosaccharides (CHOS) released by *Um*GH18A from α-chitin (A) and β-chitin (B).** All reactions were carried out in sodium citrate buffer (50 mM, pH 5.0), under stirring (1000 rpm), at 40°C using substrate (10 g.L^-1^) and *Um*GH18A (1 µM). Data points show average values and the error bars indicate standard deviations from three independent replicates.

**
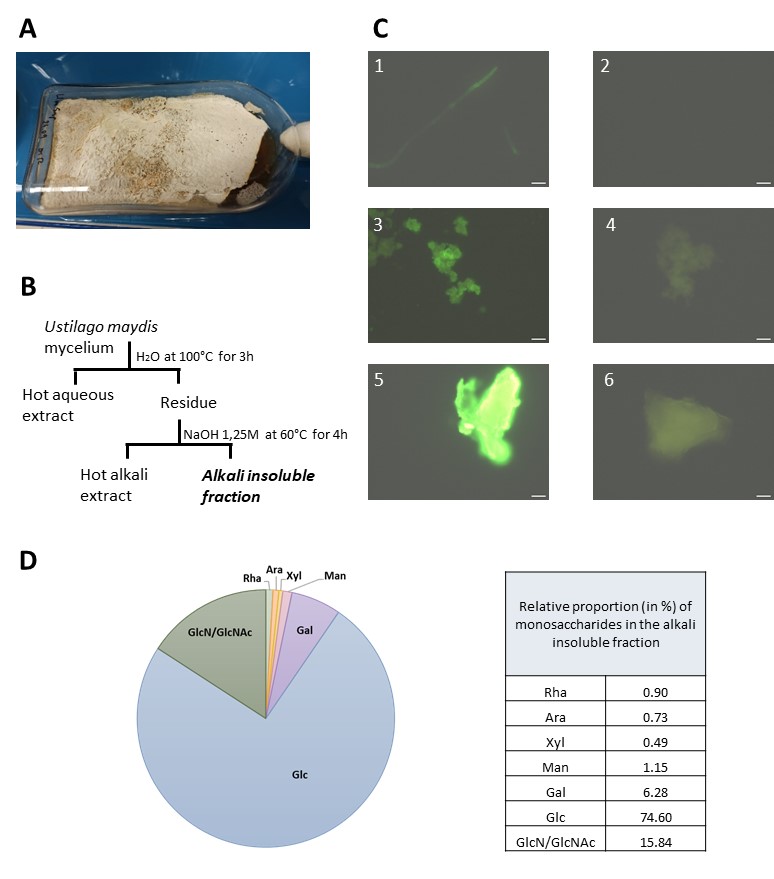
**

**Figure S9. Preparation and analysis of the *Ustilago maydis* chitin-containing fraction.** (A) Mycelium of *U. maydis* in Roux flask after 17 days of growth. (B) Outline of the protocol developed to prepare a chitin-containing fraction from *U. maydis* mycelium using sequential water and alkali extraction. (C) Fluorescence microscopy images (500X magnification, scale bar: 10µm) of *U. maydis* intact mycelium (1-top), alkali insoluble *Um*FCW fraction (3-middle) or α-chitin (5-bottom) stained with the fluorescent chitin-specific lectin WGA-AF488, and their corresponding unstained controls (Images 2, 4 and 6 of the right-hand side). (D) Monosaccharide (neutral sugars and glucosamine (GlcN/GlcNAc)) composition of the alkali insoluble fraction after acid hydrolysis.

**Additional references**

1. Mészáros B, Erdős G, Dosztányi Z. 2018. IUPred2A: context-dependent prediction of protein disorder as a function of redox state and protein binding. Nucleic Acids Research 46:W329–W337.

2. Varadi M, Anyango S, Deshpande M, Nair S, Natassia C, Yordanova G, Yuan D, Stroe O, Wood G, Laydon A, Žídek A, Green T, Tunyasuvunakool K, Petersen S, Jumper J, Clancy E, Green R, Vora A, Lutfi M, Figurnov M, Cowie A, Hobbs N, Kohli P, Kleywegt G, Birney E, Hassabis D, Velankar S. 2022. AlphaFold Protein Structure Database: massively expanding the structural coverage of protein-sequence space with high-accuracy models. Nucleic Acids Research 50:D439–D444.

3. Crooks GE, Hon G, Chandonia J-M, Brenner SE. 2004. WebLogo: a sequence logo generator. Genome Res 14:1188–1190.

4. Martin EW, Holehouse AS, Grace CR, Hughes A, Pappu RV, Mittag T. 2016. Sequence Determinants of the Conformational Properties of an Intrinsically Disordered Protein Prior to and upon Multisite Phosphorylation. J Am Chem Soc 138:15323–15335.

5. Bissaro B, Várnai A, Røhr ÅK, Eijsink VGH. 2018. Oxidoreductases and Reactive Oxygen Species in Conversion of Lignocellulosic Biomass. Microbiology and Molecular Biology Reviews 82:e00029-18.

6. Bennati-Granier C, Garajova S, Champion C, Grisel S, Haon M, Zhou S, Fanuel M, Ropartz D, Rogniaux H, Gimbert I, Record E, Berrin J-G. 2015. Substrate specificity and regioselectivity of fungal AA9 lytic polysaccharide monooxygenases secreted by *Podospora anserina*. Biotechnol Biofuels 8:90.
